# Ulysses spacecraft *in situ* detections of cometary dust trails

**DOI:** 10.1098/rsta.2023.0200

**Published:** 2024-06-23

**Authors:** Harald Krüger, Peter Strub, Eberhard Grün

**Affiliations:** ^1^ Max-Planck-Institut für Sonnensystemforschung, Göttingen, Germany; ^2^ Institut für Raumfahrtsysteme, Universität Stuttgart, Gottingen, Germany; ^3^ Max-Planck-Institut für Kernphysik, Heidelberg, Germany; ^4^ LASP, University of Colorado, Boulder, CO, USA

**Keywords:** Ulysses, interplanetary dust, cometary dust trails, IMEX, space dust

## Abstract

The Ulysses spacecraft was launched in 1990 and, after a Jupiter swing-by in 1992, became the first interplanetary spacecraft orbiting the Sun on a highly inclined trajectory with an inclination of $79^{\circ }$ . The spacecraft was equipped with an impact ionization dust detector which provided 17 years of *in situ* dust measurements in interplanetary space from 1990 to 2007. Cometary meteoroid streams (also referred to as trails) exist along the orbits of comets, forming fine structures of the interplanetary dust cloud. We use the Interplanetary Meteoroid Environment for eXploration (IMEX) dust streams in space model (Soja RH *et al*. 2015 Characteristics of the dust trail of 67P/Churyumov-Gerasimenko: an application of the IMEX model. *Astron. Astrophys.* **583**, A18. (doi:10.1051/0004-6361/201526184)) to predict cometary stream traverses by Ulysses and re-analyse the Ulysses dust dataset in order to identify impacts of cometary stream particles detected during such trail traverses. We identify 19 particles compatible with three Ulysses trail traverses on 12 March 1995, 25–27 April 2001 and 16–19 May 2001. The particle origin is compatible with up to five comets, i.e. 10P/Tempel 2, 146P/Shoemaker-LINEAR, 267P/LONEOS and possibly 45P/Honda-Mrkos-Pajdušáková and P/1999 RO28 (LONEOS). We find a dust spatial density in these trails of approximately $2-7\cdot 10^{-8}\;m^{-3}$ . The radii of the detected cometary stream particles derived from the dust instrument calibration are in the micrometre range. The *in situ* analysis of meteoroid trail particles in space, which can be traced back to their source bodies, opens a new opportunity for remote compositional analysis of comets and asteroids without the necessity to send a spacecraft to or even land on these celestial bodies, opening new opportunities for future space missions equipped with *in situ* dust analyzers.

This article is part of the theme issue 'Dust in the Solar System and beyond'.

## 1. Introduction

When a comet approaches the Sun, sublimating gases carry solid particles away from the comet’s surface. Solar radiation pressure quickly moves the small submicrometre-sized particles away from the comet nucleus, and the particles form the comet’s dust tail. Larger dust particles with sizes exceeding approximately 10μm are ejected from the cometary nucleus at lower speeds and remain very close to the comet orbit for several revolutions around the Sun [1,2]. They slowly spread along the comet’s orbit as a result of small differences in orbital period, forming a tubular structure along the orbit of the parent comet filled with dust. These structures are called meteoroid streams or dust trails. If the comet continuously releases particles over several revolutions around the Sun, they can form a visible trail that appears to follow the source comet along its orbit.

Meteoroid streams form a fine structure superimposed on the interplanetary background dust cloud. Short-term enhancements of the zodiacal light were tentatively ascribed to the optical detection of meteoroid streams [3], while cometary dust trails were first observed by the Infrared Astronomical Satellite (IRAS) [4,5] and were notable for their long, narrow appearance—sometimes extending 10s of degrees across the sky while having a width of only a few arcminutes. In a subsequent survey with the Spitzer Space Telescope in the infrared, at least 80% of the observed Jupiter-family comets were found to be associated with dust trails [6], and a ground-based survey revealed several trails also in the visible wavelength range [7]. Hence, dust trails are now considered one of the comet's common features. A recent review of cometary dust, including dust trails, was given in [8].

When the Earth intercepts a cometary trail, the particles can collide with the atmosphere and show up as meteors and fireballs, generating a meteor shower [9] (and references therein). Effects of meteoroid impacts were also observed on the Moon [10] and on other planets (see [11] for a comprehensive review). Meteoroid trails may impose a severe risk to Earth-orbiting and interplanetary spacecraft. Although the impact probability for an individual spacecraft is small, spacecraft anomalies very likely related to meteoroid impacts were observed in the past [12,13]. Hence, the risk is considered sufficiently high to occasionally affect crewed spaceflight operations [14].

Although such hazardous effects were seen in Earth orbit, the detection of meteoroid streams by *in situ* dust detectors flown on interplanetary spacecraft was hindered by the relatively small sensitive area of the instruments and low particle fluxes in the streams. Only recently could a few impacts of likely cometary meteoroid stream origin be identified in the dataset collected in the 1970s by the *in situ* dust instruments onboard the Helios spacecraft [15,16]. The aim of this article is to extend this analysis to the longest continuous dataset of *in situ* interplanetary dust measurements presently available [17] (and references therein). To this end, we re-analyse the dust measurements obtained by the Ulysses spacecraft between 1990 and 2007 and search for detections of cometary dust trail particles.

A prerequisite for this task is a newly developed tool to predict cometary meteoroid trail traverses by spacecraft. Meteoroid trails can be simulated with the Interplanetary Meteoroid Environment for eXploration (IMEX) dust streams in the space model [1,18,19]. IMEX is a universal and physical model for dust dynamics and orbital evolution of cometary dust streams in the inner Solar System. The model follows the trails of 420 comets, and it is ideal for studying meteor streams and cometary dust trails. It was developed under contract by the European Space Agency. We use the IMEX model to search for cometary meteoroid stream traverses by the Ulysses spacecraft.

We would like to point out that the two phenomena tail and trail must not be confused. Detections of ion tail passages of at least three comets were reported for Ulysses [20]. Given that the (ion and dust) tails of a comet dominantly evolve under the influence of the solar wind and solar radiation pressure, respectively, these two structures point away from the comet nucleus in the approximate anti-Sunward direction. On the other hand, the motion of the much larger trail particles is dominated by gravity. This leads to a spread of the particles along the cometary orbit over much longer timescales of many orbital periods (often several centuries) and, thus, usually in a vastly different region of space. Therefore, any coincident *in situ* detection of trails and tails far away from the comet nucleus is extremely unlikely.

In §2, we briefly describe the Ulysses mission and the dust instrument on board, and in §3, we give an overview of the IMEX model. We present the results of our IMEX simulations and compare them with the Ulysses measurements in §4. Section 5 is a discussion, and in §6, we summarize our conclusions.

## 2. Ulysses mission and dust measurements

### (a) Mission overview

Ulysses was launched on 6 October 1990 and was very successfully operated until 29 June 2009. It was the only spacecraft so far that left the ecliptic plane and passed over the poles of the Sun. The dust detector on board has been continuously operated since launch until early 2002, when—owing to the decreasing power output of the radio-isotope thermoelectric generators—individual instruments had to be turned off for intermittent periods of time to reduce power consumption. After 30 November 2007, the dust instrument remained switched off permanently.

**Figure 1.**
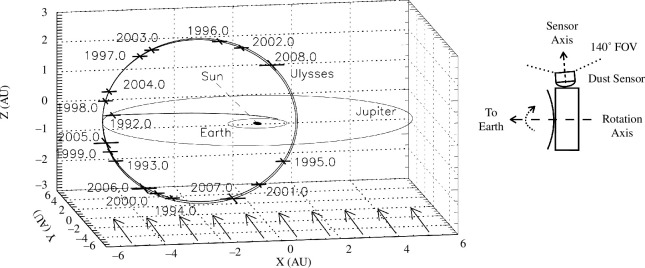
Trajectory of Ulysses in ecliptic coordinates with the Sun in the centre. The orbits of Earth and Jupiter indicate the ecliptic plane, and the initial trajectory of Ulysses was in this plane. After Jupiter flyby in early 1992, the orbit was almost perpendicular to the ecliptic plane ( 79° inclination). Crosses denote the spacecraft’s position at the beginning of each year. Vernal equinox is to the right (positive *x*-axis). The Ulysses spacecraft and the scan orientation of the dust detector are sketched for a spacecraft position close to perihelion.

Figure 1 shows the Ulysses trajectory. During a flyby at Jupiter on 8 February 1992 Ulysses was deflected on to an orbit almost perpendicular to the ecliptic plane ( $79^{\circ }$ inclination). The aphelion of Ulysses was at about 5.4 AU heliocentric distance and the perihelion distance at 1.4 AU.

The Ulysses spacecraft had a highly sensitive impact ionization dust detector on board which measured impacts of micrometre and sub-micrometre dust grains [21]. The detector was practically identical to the dust instrument flown on board the Galileo space probe between 1989 and 2003 [22].

Ulysses was a spinning spacecraft (spin period five revolutions per minute), and the dust sensor orientation at the time of a dust particle impact was recorded, allowing for a determination of the grain impact direction. The spacecraft spin axis was the centre line of its high gain antenna which normally pointed towards Earth. Most of the time the spin axis pointing was within a few degrees of the Earth’s direction, and these small deviations are usually negligible for the analysis of measurements with the dust detector. The Ulysses spacecraft and mission are explained in more detail by [23]. Details about the data transmission to Earth and the analysis of the Ulysses dust data were described by [24,25], and the data were presented in several publications [17] (and references therein).

### (b) Ulysses dust detector

The Ulysses dust detector had a $140^{\circ }$ wide field of view and was mounted on the spacecraft nearly at right angle ( $85^{\circ }$ ) with respect to the antenna axis (spacecraft spin axis). Owing to this mounting geometry, the sensor was most sensitive to particles approaching from the plane perpendicular to the spacecraft-Earth direction. The impact direction of dust particles was measured by the rotation angle, which was the sensor viewing direction at the time of a dust impact. During one spin revolution of the spacecraft, the rotation angle scanned through a complete circle of $360^{\circ }$ . Zero degrees rotation angle was defined as the direction closest to the ecliptic north. At high ecliptic latitudes, however, the sensor pointing at $0^{\circ }$ rotation angle significantly deviated from the actual north direction. Therefore, during the passages over the Sun’s polar regions, the sensor always scanned through a plane tilted by about $30^{\circ }$ from the ecliptic plane and all rotation angles lie close to the ecliptic plane (cf. figure 4 in [26]). On the contrary, during ecliptic plane crossings of Ulysses, the rotation angle approximately scanned perpendicular to the ecliptic plane, i.e. along the ecliptic latitude. A sketch of the viewing geometry can be found in [27].

**Figure 2.**
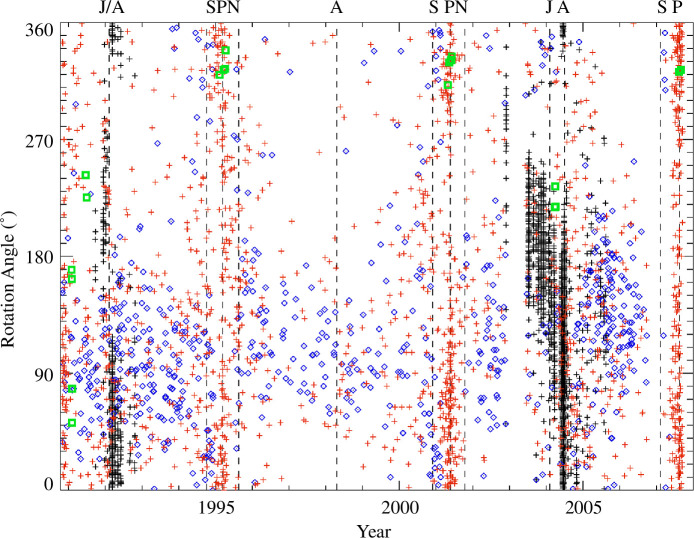
Impact direction (rotation angle) versus time for all dust impacts detected by Ulysses between launch in October 1990 and the end of dust instrument operation in November 2007. Each symbol indicates an individual dust particle impact. Red crosses: Interplanetary dust; black crosses: Jupiter stream particles [28,29]; blue diamonds: interstellar dust particles [27]. Vertical dashed lines and labels at the top indicate Jupiter flybys (**J**), perihelion passages (**P**), aphelion passages (**A**), south polar passes (**S**) and north polar passes of Ulysses (**N**). Green squares indicate the impact directions of cometary trail particles as simulated with the IMEX model (see §3).

Given that the antenna axis always pointed towards the Earth while the spacecraft moved around the Sun, the rotation angle cannot be transferred into any single celestial coordinate for the entire mission. In addition, there are also practical reasons for using the rotation angle instead of other coordinate systems. As a one-dimensional angle (as opposed to two-dimensional sky coordinates), it facilitates plotting over time. While it would be possible to achieve something similar by using an angle in a different coordinate system, such as a $360^{\circ }$ extension of the ecliptic latitude, it would also have certain disadvantages. The full range of rotation angles would only cover a variable fraction of the other angular coordinate, depending on the orbital geometry, and would lead to a variable line element in the transformation from rotation angle to another coordinate system. In the plot showing the rotation angle over time (figure 2), variations in measured dust flux can easily be identified just from the density of points. After a transformation to other coordinate systems, this would no longer be possible, as the visual density in the plot can be reduced or enhanced in the coordinate transformation.

Figure 2 shows the rotation angle at the time of a particle impact. The colours highlight different populations of dust identified in the Ulysses dust data so far: (i) Interstellar dust particles which enter the heliosphere from the upstream direction of the Sun’s motion through the ambient interstellar medium and sweep through the Solar System (shown in blue [30]); (ii) interplanetary particles originating from the zodiacal dust cloud (shown in red [26]); and (iii) streams of dust particles emanating from the Jupiter system which appear as vertical stripes in 1992 and in 2003–2005 (shown in black [29,31]). The latter are ejected from the Jupiter system by their electromagnetic interaction with the planet’s magnetic field. More details about these dust populations studied with Ulysses can be found in [17] (with references given therein).

The Ulysses dust detector was a multi-coincidence impact-ionization detector. Upon impact of a single dust particle on to its sensor target, it measured up to three charge signals in a multi-coincidence measurement: negatively charged ions and electrons were measured at the detector target, positively charged ions at an ion collector grid, and a second positive charge signal was measured at a channeltron [21]. From the charge measured at the ion collector grid, the particle impacts were categorized into six amplitude ranges (AR). Each AR covers an order of magnitude in impact charge. To first order, for a constant impact speed, these AR values are directly related to the particle mass, covering six orders of magnitude in mass. Therefore, in the analysis of the dust data, AR can be considered as an approximate indicator of particle mass. For details, the reader is referred to [24].

### (c) Dust measurements

For each event registered on the sensor target (dust particle impact or noise event), the three charge signals together with measurements of the charge rise times and coincidences of the charge signals provide a classification of the measured events into four different quality classes [24,32] (the classification scheme was changed in 2002, but this is not relevant for our analysis presented here). A larger number of measured charge signals in combination with appropriate coincidences implied a higher likelihood of a real dust impact. Noise events were assigned to the lower-quality classes while the higher classes are usually considered noise-free. From laboratory calibrations and measurements in space, we have gained a good understanding of the noise behaviour of the instrument since launch.

For the analysis described in this article, we have used the (noise-removed) dust dataset as published in [17] (and references therein). We have also used the knowledge from our twin dust instrument on board the Galileo spacecraft which was orbiting Jupiter from 1995 to 2003 [22] to optimize our understanding of the noise behaviour of both instruments, e.g. [33] (and references therein). The noise identification was discussed in detail in [28] and [34].

The overall dust impact rates measured by the Ulysses dust detector during the entire mission from all rotation angles averaged over a three-month time interval, varied between approximately 0.3 impacts per day at high ecliptic latitudes and maximum values of about 1.5 per day in the inner Solar System around the spacecraft’s perihelion passages close to the ecliptic plane [35]. At high latitudes, most of the detected impacts were owing to particles of interstellar origin, only a minor fraction being interplanetary particles. In contrast, in the inner Solar System, the majority of impacts were interplanetary particles, in fact at perihelion passage the interplanetary particles outnumber the interstellar ones by about a factor of three. The Ulysses dust dataset is the largest set of continuous dust measurements in interplanetary space recorded by a dedicated dust detector available to date.^1^

## 3. Cometary dust trail simulations

### (a) IMEX cometary dust trails model

In order to identify time intervals when Ulysses traversed cometary meteoroid trails, we use the IMEX dust streams in the space model [1,18,19]. The model generates trails for 362 Jupiter-family, 40 Halley-type and 18 Encke-type comets available in the JPL Small-Body Database (SBDB) as of 1 August 2013, which have perihelion distances q < 3 AU, semimajor axes a < 30 AU and defined total visual magnitudes.

Particles are emitted when a comet is in the inner Solar System, taking into account perihelion passages between the years 1700 and 2080 for Encke-type comets, and between 1850 and 2080 for Jupiter-family and Halley-type comets, respectively. This reflects the fact that the most recently released dust is expected to be the most important, and also the maximum size of the database that could be maintained at the time when the model was developed.

For each comet and for each passage through the inner Solar System within 3 AU of the Sun (which we refer to as apparition in the following), particles are emitted from the sunlit hemisphere of the comet nucleus within the time ranges specified above. About 28 000 particles are ejected per comet per apparition for Halley-type comets and about 14 000 for other comets.

The dust ejection is described by the comet emission model of [36]. The model assumes the dust emission to be driven by water gas production within 3 AU of the Sun. It estimates the water production rate using the visual magnitude, and a gas-to-dust ratio [37]. The JPL SBDB provides total and nuclear magnitudes.

Dust-to-gas mass ratios can be estimated for individual comets, and they mostly range from 0.1 to 3, though higher values are possible. They appear to be dependent on heliocentric distance [38]. Given the large uncertainties in dust-to-gas ratios, the model uses a value of 1. Deviations from this value can be considered in the analysis of individual comets.

The IMEX model uses the mass distribution of [39] and [2,40], with model parameters given by [1]. The mass distribution covers the range from $10^{-8}\;$ to $10^{-2}\;kg$ , separated into eight mass bins (approximately corresponding to 100μm to 1 cm particle radius [1]). The particle density is assumed to be $\rho =1000\;kg\;m^{-3}$ . For comet nuclei with unknown radius, a value of 1 km is assumed [1].

The trajectory of each emitted particle is integrated individually including solar gravity, planetary perturbations, solar radiation pressure and Poynting–Robertson drag. The model calculates the impact velocity for each individual particle onto the spacecraft as well as dust number density and flux. A detailed model description including an application to the trail of comet 67P/Churyumov–Gerasimenko was given by [1].

The IMEX model was successfully applied to identify a small number of likely cometary trail particle impacts in the *in situ* dust dataset measured by the Helios spacecraft [16]. Furthermore, the model was used to predict future dust trail traverses by the Bepi Colombo spacecraft en route to Mercury [41] and by the Martian Moons Exploration (MMX) mission to Phobos and Deimos [42] to be launched in 2024.

### (b) Simulation results

#### (i) Trail traverses and dust fluxes

**Figure 3.**
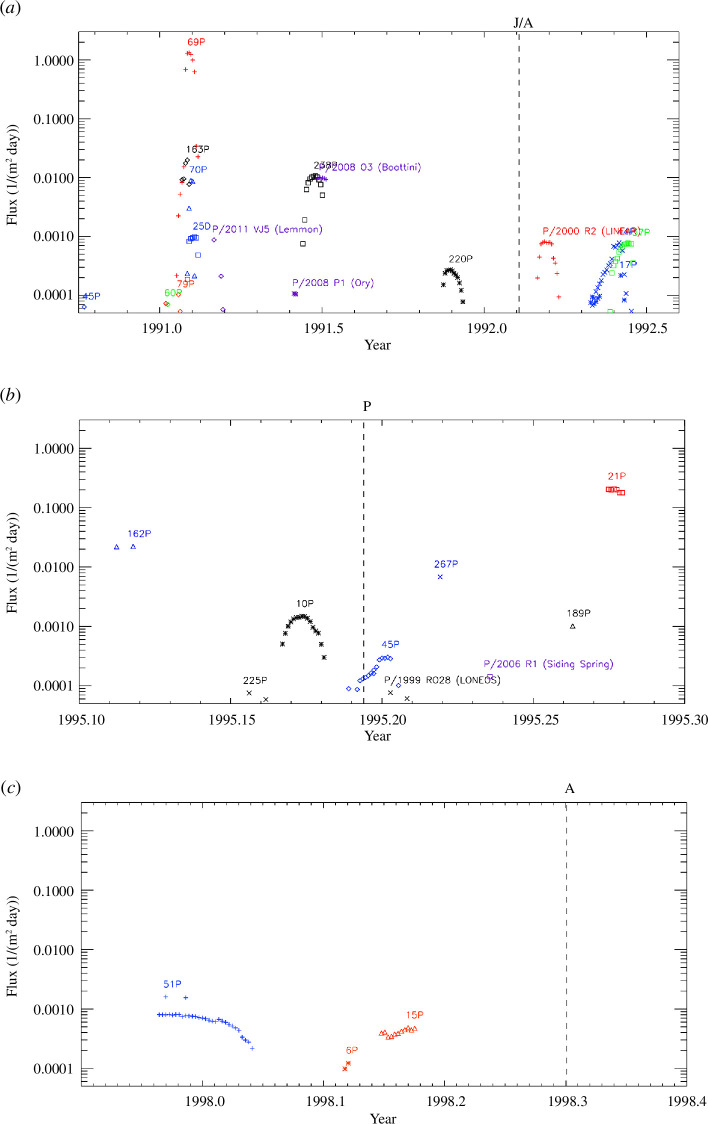
Simulated dust fluxes for cometary meteoroid streams intercepted by the Ulysses spacecraft with fluxes exceeding 5⋅10^−5^ m^−2^ day^−1^. The simulated particles are greater than or equal to 100 μm . The simulated data points are separated by 2 days, and colours and symbols distinguish individual comets. The meaning of the vertical dashed lines is the same as in figure 2.

**Figure 4.**
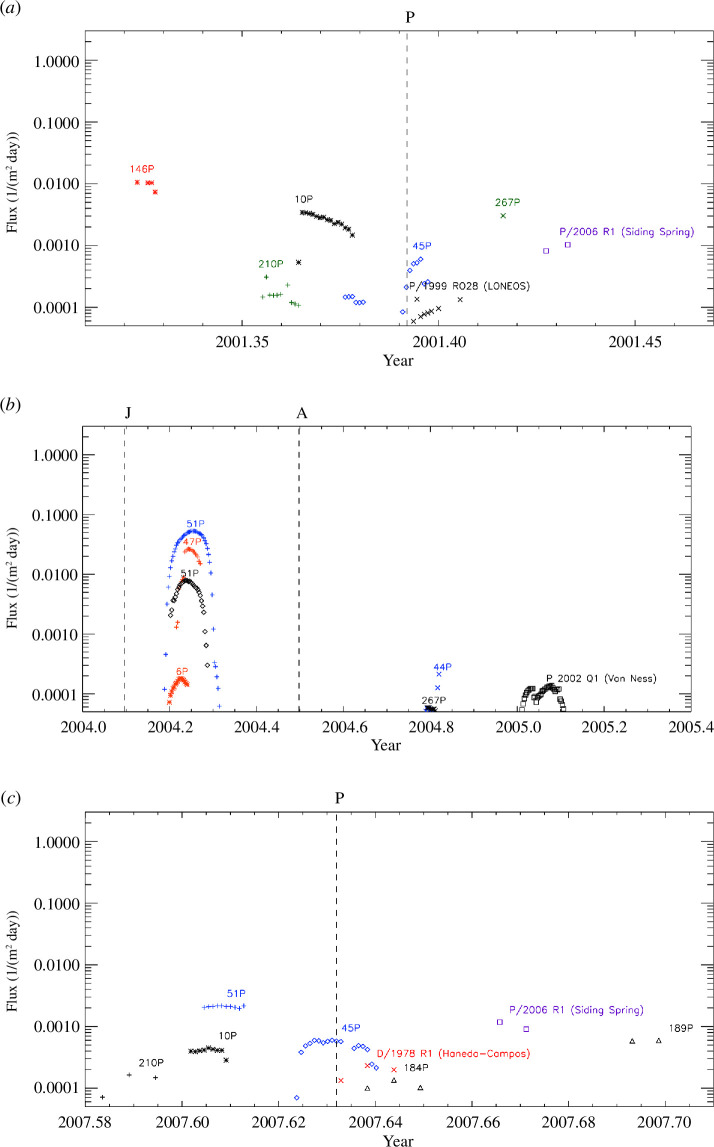
Same as figure 3 but for the second part of the Ulysses mission from 2001 to 2007.

The results of our IMEX simulations for the entire Ulysses mission are shown in figures 3 and 4. The model predicts cometary trail traverses only close to the ecliptic plane, while at higher latitudes, it does not predict any traverse. Such a gap at high latitudes is not surprising because almost 90% of the comets used in the model are Jupiter family comets, which have low orbital inclinations, while highly inclined Halley-type comets make up only 10% of the model database. No trail traverses were predicted for time intervals not covered by these diagrams.

For the entire operational period of the Ulysses dust instrument from 1990 to 2007, the model identified a total of 80 cometary trail traverses. For the majority of these traverses, the predicted dust fluxes are below $10^{-3}\;m^{-2}\;day^{-1}$ . For our further analysis, we preferentially consider trail traverses with predicted fluxes above this limit. At first glance, this flux limit looks very low. However, IMEX only simulates particles with sizes 100μm and bigger, while the Ulysses dust instrument measured particles that were at least one order of magnitude smaller and—given the particle size distribution increasing towards smaller particles—are expected to be much more abundant in the dust stream. Furthermore, a limit in this range is also indicated by our earlier analysis of the Helios *in situ* dust data in combination with IMEX model results [16]. We shall get back to this in §5. Nevertheless, this limit leaves us with 19 candidate trail traverses, which are listed in table 1. The orbital elements of the corresponding comets are given in table 5, in appendix A.

**Table 1.** Results from the IMEX cometary dust trail simulations for Ulysses for trail traverses with predicted fluxes exceeding $10^{-3}\;m^{-2}\;day^{-1}$ , except the three bottom ones which have lower fluxes. The time interval of the trail traverses together with the predicted maximum flux (of 100μm and bigger particles) are given in columns (2)–(5). Column (6) gives the fluence to a $1\;m^{2}$ detector predicted for the time interval given in column (4). Comets with likely trail particle detections by Ulysses as derived from our analysis described in §4 are written in italics.

comet (1)	day max. flux (dd.mm.yyyy) (2)	time interval with flux $>10^{-3}\;m^{-2}\;day^{-1}$ (dd.mm.yyyy) (3)	duration (days) (4)	max. flux $\left(m^{-2}\;day^{-1}\right)$ (5)	fluence $\left(m^{-2}\right)$ (6)
163P/NEAT	31.01.1991	25.01.1991–02.02.1991	9	0.02	0.1
69P/Taylor	02.02.1991	21.01.1991–12.02.1991	23	1.3	7.1
70P/Kojima	04.02.1991	02.02.1991–06.02.1991	5	0.009	0.03
25D/Neujmin 2	06.02.1991	06.02.1991–07.02.1991	2	0.001	0.002
238P/Read	20.06.1991	12.06.1991–02.07.1991	21	0.01	0.17
304P/Ory	30.06.1991	26.06.1991–06.07.1991	11	0.01	0.11
162P/Siding-Spring	10.02.1995	10.02.1995–12.02.1995	3	0.02	0.05
*267P/LONEOS*	*21.03.1995*	*21.03.1995*	*1*	*0.007*	*0.007*
189P/NEAT	06.04.1995	06.04.1995	1	0.001	0.001
21P/Giacobini-Zinner	11.04.1995	10.04.1995–11.04.1995	2	0.2	0.4
*146P/Shoemaker-LINEAR*	*29.04.2001*	*28.04.2001–29.04.2001*	*2*	*0.01*	*0.02*
*10P/Tempel 2*	*13.05.2001*	*13.05.2001–18.05.2001*	*6*	*0.003*	*0.008*
267P/LONEOS	01.06.2001	01.06.2001	0.5	0.003	0.006
383P/Christensen^a^	07.06.2001	07.06.2001	1	0.001	0.001
51P/Harrington-A	26.03.2004	14.03.2004–12.04.2004	29	0.007	0.1
47P/Ashbrook–Jackson	29.03.2004	19.03.2004–08.04.2004	20	0.02	0.2
51P/Harrington	04.04.2004	11.03.2004–19.04.2004	40	0.05	0.8
51P/Harrington	11.08.2007	09.08.2007–11.08.2007	3	0.02	0.06
383P/Christensen^a^	31.08.2007	31.08.2007–01.09.2007	2	0.001	0.002
45P/Honda–Mrkos–Pajdušáková	15.03.1995	—	(2)	0.0003	0.0005
45P/Honda–Mrkos–Pajdušáková	24.05.2001	—	(1)	0.0006	0.0005
P/1999 RO28 (LONEOS)	28.05.2001	—	(2)	0.0002	0.0001

^a^ Other designation (P 2006 R1 Siding Spring).

Figures 3 and 4 show the highest flux for the traverse of the trail of comet 69P/Taylor in January 1991 with a simulated flux of $1.3\;m^{-2}\;day^{-1}$ . The corresponding fluence during the entire trail traverse which lasts 23 days is seven particles bigger than 100μm detectable per square metre (table 1). The second highest flux of $0.2\;m^{-2}\;day^{-1}$ is predicted for comet 21P/Giacobini–Zinner for April 1995. For four additional comets 163P/NEAT, 162P/Siding-Spring, 47P/Ashbrook–Jackson and 51P/Harrington the predicted fluxes exceed $10^{-2}\;m^{-2}\;day^{-1}$ .

Unfortunately, the spacecraft orientation and the field of view of the Ulysses dust detector prevented the detection of particles originating from all these comets with relatively high fluxes. Only particles from several comets with lower fluxes were detectable based on the detection geometry as we shall show below.

#### (ii) Particle impact direction

In order to reliably identify dust particle impacts originating from cometary trails in the Ulysses dust dataset, we have to consider the dust sensor detection geometry in relation to the particle approach direction predicted by the model. The detection geometry is determined by the $140^{\circ }$ wide dust sensor field of view and the orientation of the spacecraft rotation axis (cf. §2.2).

**Figure 5.**
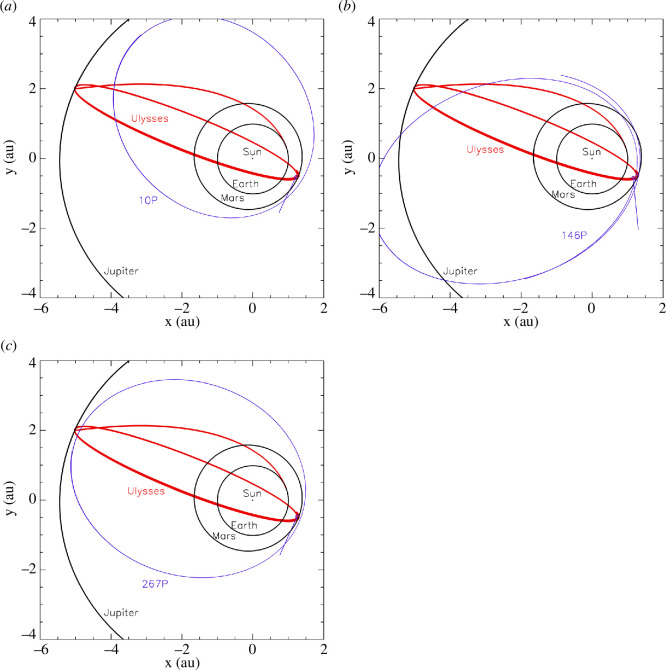
Interplanetary trajectory of Ulysses (red line) together with cometary orbits (blue lines), which had a favourable geometry for trail particle detection, and dust trail particles were likely identified in the Ulysses dataset (see §4). Blue bars indicate the approach direction (velocity vector) of trail particles on to the spacecraft in the spacecraft-centred reference frame projected onto the X–Y plane (cf. table 1). The X–Y plane is the ecliptic plane with vernal equinox oriented towards the +X direction.

To illustrate the geometrical conditions for trail particle detections, we show in figure 5 the Ulysses orbit projected on to the ecliptic plane together with the orbits of three individual comets. We shall show in §4 that Ulysses likely detected trail particles from these comets during traverses of their trails. All three comets are Jupiter Family Comets orbiting the Sun in a prograde sense with inclinations up to $23^{\circ }$ (cf. table 5). The impact directions of trail particles are indicated in the spacecraft-centred frame as derived from the model, and they are also listed in table 2.

**Table 2.** Dynamical properties of the Ulysses cometary dust trails traverses predicted by the IMEX cometary dust trails simulations. Columns (3) and (4) give the impact direction of the particles in ecliptic coordinates (opposite to the velocity vector) while columns (5)–(8) give the velocity vector, both are given in the spacecraft-centred reference frame. The impact direction (columns (3) and (4)) corresponds to the radiant of a meteor stream in a planetary atmosphere. The uncertainty ranges in columns (5)–(8) give the 1σ variation of the simulated impact velocities.

comet (1)	day (dd.mm.yyyy) (2)	$\lambda_{ecl}$ ${(}^{\circ })$ (3)	$\beta_{ecl}$ ${(}^{\circ })$ (4)	$v_{imp}$ $\left(km\;s^{-1}\right)$ (5)	$v_{x}$ $\left(km\;s^{-1}\right)$ (6)	$v_{y}$ $\left(km\;s^{-1}\right)$ (7)	$v_{z}$ $\left(km\;s^{-1}\right)$ (8)
163P/NEAT	31.01.1991	98	−17	17.6±0.6	2.4±0.2	−16.7±0.5	5.1±0.2
69P/Taylor	02.02.1991	104	−24	20.5±0.5	4.5±0.3	−18.1±0.5	8.5±0.1
70P/Kojima	04.02.1991	102	9	22.3±0.6	4.7±0.2	−21.5±0.5	−3.6±0.2
25D/Neujmin 2	06.02.1991	108	6	27.1±0.8	8.2±0.3	−25.7±0.4	−2.8±0.6
238P/Read	20.06.1991	128	1	13.6±0.3	8.3±0.2	−10.8±0.2	−0.1±0.0
304P/Ory	30.06.1991	129	−2	8.6±0.3	5.4±0.2	−6.7±0.2	0.3±0.0
162P/Siding-Spring	10.02.1995	280	36	32.5±0.7	−4.7±0.5	26.0±0.4	−19.0±0.3
*267P/LONEOS*	*21.03.1995*	*246*	*53*	42.5±0.8	10.4±0.7	23.4±0.4	−33.9±0.2
189P/NEAT	06.04.1995	223	42	35.1±0.8	19.0±0.6	17.8±0.5	−23.6±0.3
21P/Giacobini-Zinner	11.04.1995	236	64	52.1±0.9	12.8±0.7	18.7±0.3	−46.9±0.3
*146P/Shoemaker-LINEAR*	*29.04.2001*	*276*	*42*	38.7±0.8	−2.9±0.7	28.6±0.3	−25.9±0.3
*10P/Tempel 2*	*13.05.2001*	*245*	*56*	45.1±0.9	10.7±0.7	22.8±0.5	−37.4±0.2
267P/LONEOS	01.06.2001	246	53	42.4±0.8	10.5±0.7	23.4±0.3	−33.9±0.2
383P/Christensen	07.06.2001	244	53	43.9±1.0	11.5±0.9	23.6±0.3	−35.2±0.2
51P/Harrington-A	26.03.2004	57	−52	11.6±0.2	−3.8±0.2	−6.0±0.1	9.2±0.0
47P/Ashbrook–Jackson	29.03.2004	65	−36	10.4±0.1	−3.6±0.1	−7.6±0.1	6.1±0.0
51P/Harrington	04.04.2004	56	−52	11.6±0.2	−4.0±0.2	−5.9±0.1	9.2±0.1
51P/Harrington	11.08.2007	258	53	41.0±1.2	5.4±0.7	24.4±0.3	−32.5±0.9
383P/Christensen	31.08.2007	242	55	43.1±0.8	11.6±0.7	22.2±0.3	−35.1±0.1
45P/Honda–Mrkos–Pajdušáková	13.03.1995	294	49	42.2±1.0	−11.2±0.8	25.4±0.6	−31.8±0.1
45P/Honda–Mrkos–Pajdušáková	24.05.2001	294	49	42.0±0.9	−11.1±0.8	25.0±0.4	−31.8±0.2
P/1999 RO28 (LONEOS)	28.05.2001	261	59	48.1±1.4	3.9±0.8	24.9±0.5	−41.0±1.0

The spacecraft rotation angles corresponding to these predicted impact directions are indicated in figure 2 as green squares, and they are listed in table 3 (column (3)). All three trail traverses with favourable detection conditions occurred in the inner Solar System close to the perihelion passages of Ulysses.

**Table 3.** Particle detection geometry derived from the IMEX model. Column (3) lists the rotation angles for trail particles, column (4) gives the rotation angle for particles on circular heliocentric orbits on the day of the flux maximum listed in column (2). Columns (5) and (6) list the minimum impact angle with respect to the sensor axis during one spacecraft revolution of trail particles and particles on circular orbits, respectively. Column (7) gives the predicted detection range in rotation angle, ΔROT , for trail particles.

comet (1)	date flux maximum (dd.mm.yyyy) (2)	$ROT_{trail}$ ${(}^{\circ })$ (3)	$ROT_{circ}$ ${(}^{\circ })$ (4)	$\theta_{min,trail}$ ${(}^{\circ })$ (5)	$\theta_{min,circ}$ ${(}^{\circ })$ (6)	$\Delta ROT_{trail}$ ${(}^{\circ })$ (7)
163P/NEAT	31.01.1991	170	251	74	88	—
69P/Taylor	02.02.1991	163	252	66	88	68
70P/Kojima	04.02.1991	52	252	85	88	—
25D/Neujmin 2	06.02.1991	78	253	82	88	—
238P/Read	20.06.1991	243	269	93	60	—
304P/Ory	30.06.1991	225	269	90	57	—
162P/Siding-Spring	10.02.1995	320	325	28	9	133
*267P/LONEOS*	*21.03.1995*	*324*	*324*	*1*	*0*	142
189P/NEAT	06.04.1995	324	327	17	3	142
21P/Giacobini-Zinner	11.04.1995	339	328	4	4	142
*146P/Shoemaker-LINEAR*	*29.04.2001*	*312*	*320*	*1*	*13*	142
*10P/Tempel 2*	*13.05.2001*	*329*	*324*	*18*	*15*	142
267P/LONEOS	01.06.2001	331	332	14	14	143
383P/Christensen	07.06.2001	334	335	12	13	143
51P/Harrington-A	26.03.2004	218	253	2	13	143
47P/Ashbrook–Jackson	29.03.2004	234	253	6	13	142
51P/Harrington	04.04.2004	218	253	1	14	142
51P/Harrington	11.08.2007	322	324	22	26	141
383P/Christensen	31.08.2007	324	322	22	21	136
45P/Honda–Mrkos–Pajdušáková	13.03.1995	326	324	30	2	132
45P/Honda–Mrkos–Pajdušáková	24.05.2001	320	329	12	15	139
P/1999 RO28 (LONEOS)	28.05.2001	331	330	5	15	143

## 4. Data analysis

In this section, we describe our analysis of the Ulysses dust data in order to identify the impacts of particles originating from cometary trails. Based on the dust densities predicted for cometary trails by the IMEX model, one might expect that clustering of impacts in the data could easily be identified when the spacecraft traversed some of the densest trails. Apart from the prominent Jupiter dust streams detected in the 1991/1992 and 2003–2005 time intervals which in many cases can be seen in the dataset even without further processing (cf. Figure 2); however, there is no obvious impact clustering. Hence, in order to identify any clustering by cometary trails we have to use more sophisticated analysis techniques.

### (a) Search for dust trail particles

Given the relatively small dust fluxes encountered in interplanetary space, we have to deal with small number statistics. Real short-time enhancements in the impact rate must be reliably separated from random fluctuations of the background flux. The method we use to identify impact clustering was first employed by [43] for impacts on the Moon, and its application to Jovian dust streams in the Ulysses dust data was described in [44].

We formed time intervals by sliding a window with a fixed number of impact events, N ( N∈{4,5,6,7} ), over the dataset and used Poisson statistics to calculate the probability, P , that these impacts are owing to a random fluctuation. The first and last particle in the window defines the time interval Δt . The probability P(N,X) for observing at least N events, where X is the number of background events expected in the interval Δt , is:


(4.1) $$P(N,X)=1-e^{-X}\sum_{m=0}^{N-1}\frac{X^{m}}{m!}.$$

A minimum in the probability function below a given value indicates a clustering of impacts which may be owing to a dust trail traverse. We chose a probability of 3σ= 99.72% in the single interval (i.e. before correcting for the family-wise error rate) as a detection threshold for a trail candidate. If the midpoint of the candidate’s time interval Δt differs from nearby simulated trails by more than 1.5Δt , the candidate is rejected. This leads to a search interval three times the size of the trail width, and we therefore have to multiply the probability by 3 to correct for the family-wise error rate. Hence, the probability that dust impacts we identify as trail particles are in reality caused by a random fluctuation is below 0.28%. We take the average background dust impact rate $r_{background}$ from the number of impacts measured in the time interval ±15 days around each of the perihelion passages of Ulysses and calculate the background particle count $X=\Delta t\cdot r_{background}$ in the time interval Δt .

With this assumption, we searched through the entire Ulysses dataset by sliding a window with a given N over the data. We started the search with N=4 , which is the lowest count that can lead to statistically significant results with the typical backgrounds in the dataset, and we varied it in a range between 4 and 7. We take this into account by multiplying the Poisson probabilities derived from the background flux by a factor of 4, leading to an overall factor for the family-wise error rate of $P_{fw}=12P$ .

This is a conservative estimate, likely overestimating the probability of a statistical fluke because it assumes that the four particle groups under consideration are statistically independent. This assumption is not fulfilled here: owing to selecting the particles for different N from the same dataset, the selection of seven particles is a superset of the four-particle selection.

As a first step, we used the entire Ulysses dust dataset, i.e. dust impacts detected with all rotation angles, ROT, or in other words, impacts detected from all possible scan directions of the sensor. The search result was negative, i.e. we did not find any clustering of particles at the chosen 3σ level, apart from the prominent Jupiter dust streams in 1991/1992 and 2004/2005 identified earlier (cf. [28,29]; see also figure 2).

Next, we took into account the restricted field of view of the dust sensor together with the impact directions of trail particles predicted by the model (cf. §3.2.2). The full sensitive area is $0.1\;m^{2}$ ; however, this value applies only to particles approaching parallel to the sensor axis (impact angle $\theta =0^{\circ }$ ). For impact angles $\theta =32^{\circ }$ with respect to the sensor axis, the effective sensor area is reduced to half its maximum value, and for $\theta =70^{\circ }$ it is 0 [22] (note that these values neglect the sensitivity of the sensor side wall as found by [45]). We therefore restricted the search range in impact directions from the full $360^{\circ }$ to a narrower range in rotation angles, $\Delta ROT=64^{\circ }$ , covering the whole range of impact angles $\pm 32^{\circ }$ where the detector’s sensitivity exceeds 50% of the maximum sensitivity, the latter applies to particles approaching parallel to the sensor axis. This restriction improves the suppression of background particles.

Table 3 lists six trail traverses for 1991. Column (6) shows that five of these traverses occurred with $\theta_{min}>70^{\circ }$ and therefore the trail particles’ approach direction was outside the field of view of the dust sensor; for the sixth comet, 69P/Taylor, the impact angle was $\theta_{min}\approx 66^{\circ }$ , and hence, the effective sensor area was very small.

The remaining 13 traverses were predicted to occur with $\theta_{min}≲28^{\circ }$ . Thus, these trails were well within the sensor’s field of view, and they were good candidates for potential trail particle identifications. All of these 13 traverses are predicted for the time interval 1995–2007, i.e. for the out-of-ecliptic part of the Ulysses mission when the spacecraft was on its highly inclined interplanetary trajectory. Three of these trail crossings were predicted for 2004 around the second Jupiter flyby of Ulysses. During its two flybys at Jupiter, the dust detector measured streams of dust particles emanating from the giant planet’s innermost Galilean moon Io. They were detected in interplanetary space out to 3.5 AU from the planet in 2003−2005 [29]. We have to ignore these three cometary trail traverses in our analysis because a reliable separation of cometary trail particles from Jupiter streams is not possible given that both types of particles approached the spacecraft from similar directions (cf. figure 2). Based on these considerations, we have a total number of 10 candidate trail traverses where the identification of particle concentrations in the Ulysses dust data appeared promising.

As a result, our statistical analysis revealed particle clustering above the 3σ level for three trail traverses. These occurred within a few days of the predicted times, and they are listed in table 4. As expected, no particle clustering was found in 1991, in agreement with the detection geometry which implied that no trail particles should be detectable. Furthermore, of the remaining 10 traverses with favourable detection conditions, 7 traverses did not show any detections in the Ulysses dataset. Our detailed analysis results are presented next.

**Table 4.** Results from the Ulysses cometary dust trail analysis. Column (2) lists the date range of the dust trail traverse derived from the measurements, and column 3 the exact time interval Δt measured for each stream from the particle impact times. column (4) gives the interplanetary background dust impact rate derived from a time interval ±15 days around the Perihelion passage of Ulysses. Column (5) lists the total number of particles identified during each trail traverse, while column (6) lists the number of particles with a reliable impact calibration. Columns (7) and (8) list the detection $\sigma_{det}$ for the single interval (i.e. uncorrected for the family-wise error rate), and $\sigma_{fw}$ using the probability corrected by the factor of 12 to account for the family-wise error rate, respectively. Columns (9) and (10) give the average particle impact speed and mass for each of the trail traverses, column (11) gives the average particle mass assuming the simulated impact speed for the mass calibration, column (12) gives the particle radius assuming a particle density of $1000\;kg\;m^{-3}$ and the mass listed in column (12). Column (13) gives the measured dust flux derived from equation (4.2), and column (14) lists the dust spatial density in the trail given by equation (4.3). See text for details.

comet (1)	stream date $t_{cross}$ (dd.mm.yyyy) (2)	stream duration (days) (3)	$r_{background}$ $\left(day^{-1}\right)$ (4)	$N_{det}$ (5)	$N_{calib}$ (6)	$\sigma_{det}$ (7)	$\sigma_{fw}$ (8)
267P/LONEOS	12.03.1995	1.44	0.200	5	3	4.36	3.78
146P/Shoem.-L.	25−27.04.2001	3.59	0.267	7	6	4.00	3.36
10P/Tempel 2	16−19.05.2001	3.20	0.267	7	5	4.16	3.55

comet (1)	$\bar{v_{calib}}$ $\left(km\;s^{-1}\right)$ (9)	$\bar{m_{calib}}$ (kg) (10)	$\bar{m_{vtheo}}$ (kg) (11)	$r_{d}$ (μm) (12)	trail flux Φ $\left(m^{-2}\;day^{-1}\right)$ (13)	density D $\left(m^{-3}\right)$ (14)
267P/LONEOS	17	$4\cdot 10^{-14}$	$4\cdot 10^{-15}$	2.1	≈267	$\approx 7\cdot 10^{-8}$
146P/Shoem.-L.	41	$1\cdot 10^{-14}$	$1\cdot 10^{-15}$	1.3	≈115	$\approx 3\cdot 10^{-8}$
10P/Tempel 2	43	$4\cdot 10^{-15}$	$8\cdot 10^{-16}$	1.0	≈82	$\approx 2\cdot 10^{-8}$

^a^ Other designation: P 2006 R1 Siding Spring.

### (b) Ulysses measurements versus IMEX simulations

**Figure 6.**
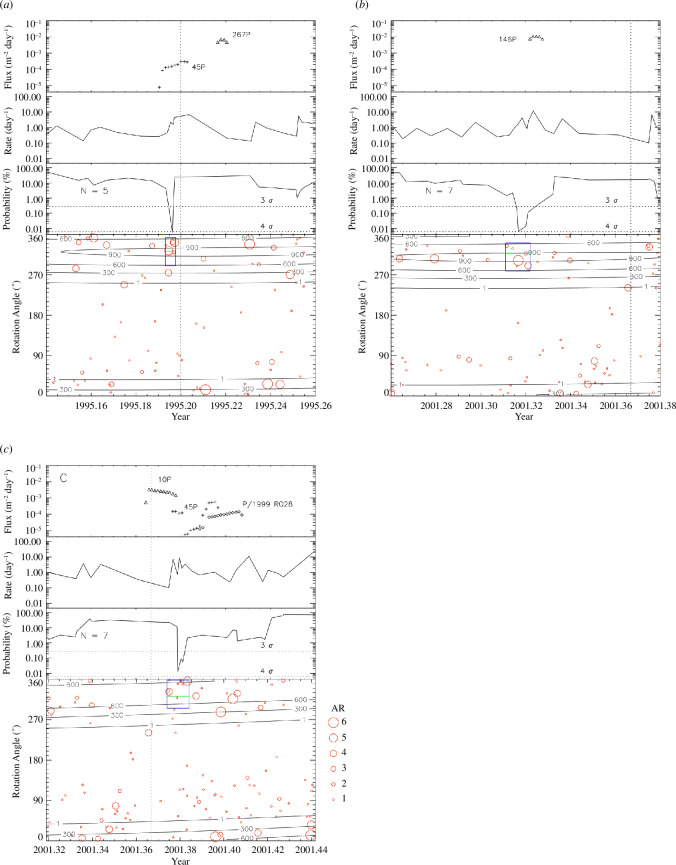
Comparison of IMEX simulations with Ulysses measurements. In each of the three sets of diagrams the top panel shows the simulated dust flux (data points are separated by 12 h, the second panel shows the impact rate measured by Ulysses, the third panel shows the probability for observing at least N events based on Poisson statistics (equation (4.1)), here the dashed horizontal lines indicate the 3 σ and 4 σ levels. The bottom panel shows the rotation angles of all particles measured in the time intervals shown. Here, the identified likely cometary trail particles are indicated by blue boxes. The horizontal green line indicates the approach direction of particles on circular prograde heliocentric orbits in this time interval, and contour lines show the effective dust sensor area for cometary stream particles approaching from the simulated direction. Vertical dotted lines indicate the times when Ulysses crossed the ecliptic plane. The symbol size indicates the amplitude range, AR, of the impact charge, see text for details. During the time intervals shown here the rotation angle approximately scanned along the ecliptic latitude (see §2.2 for details).

**Figure 7. AF7:**
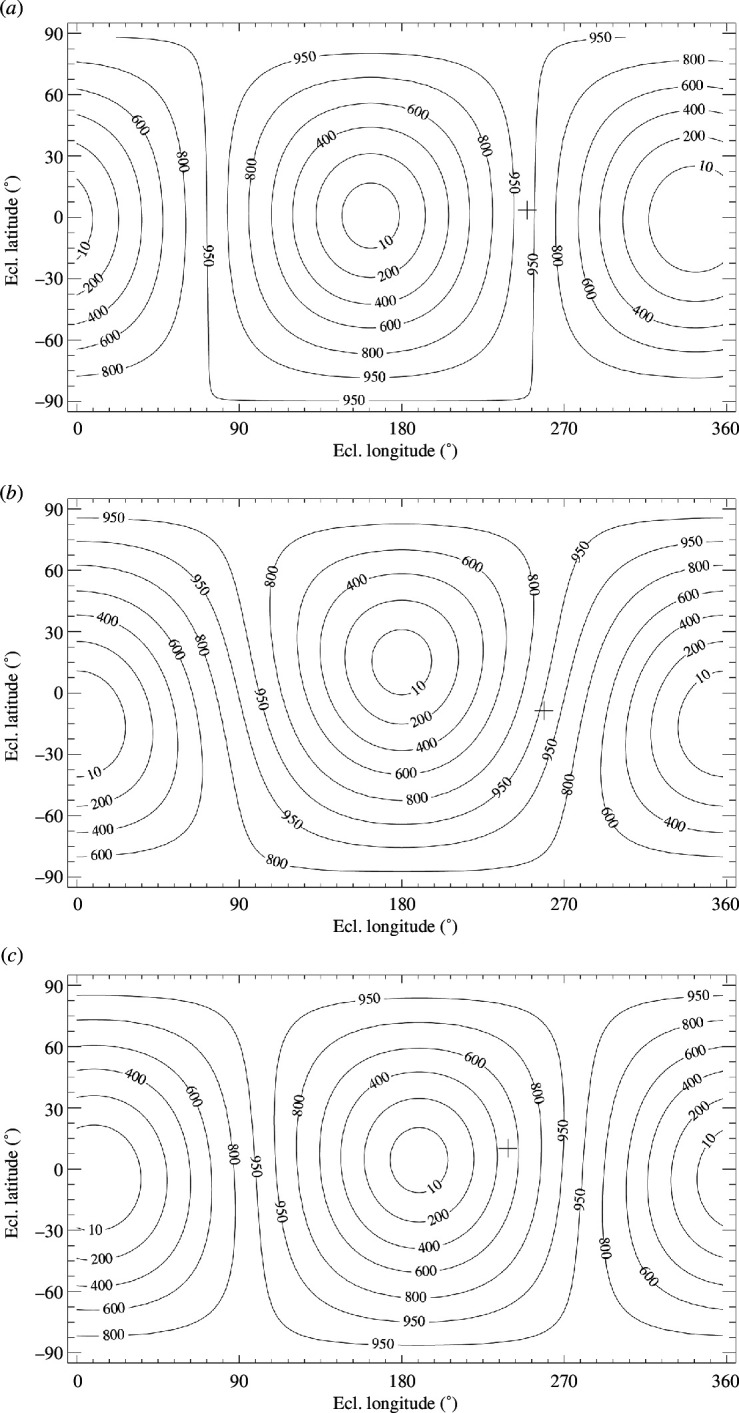
Sky maps of the Ulysses dust detector’s sensitive area owing to the detectors’ scanning pattern in the spacecraft frame for the thre dust stream detections reported in this work. We use the spacecraft position and velocity vector on the date for each stream detection for calculating the transformation between the solar system barycentric reference frame and the spacecraft frame. The ‘+’ sign denotes the direction of the stream in the spacecraft frame as predicted by the IMEX Streams model. *Top panel:* Stream detection on 12 March 1995, with a stream velocity of 31.8 km s^−1^ as predicted by the IMEX Streams model. *Middle panel:* Detection on 25–27 April 2001, and a stream velocity of 32.2 km s^−1^. *Bottom panel:* Detection on 16–19 May 2001, and a stream velocity of 32.1 km s^−1^ .

**Figure 8.**
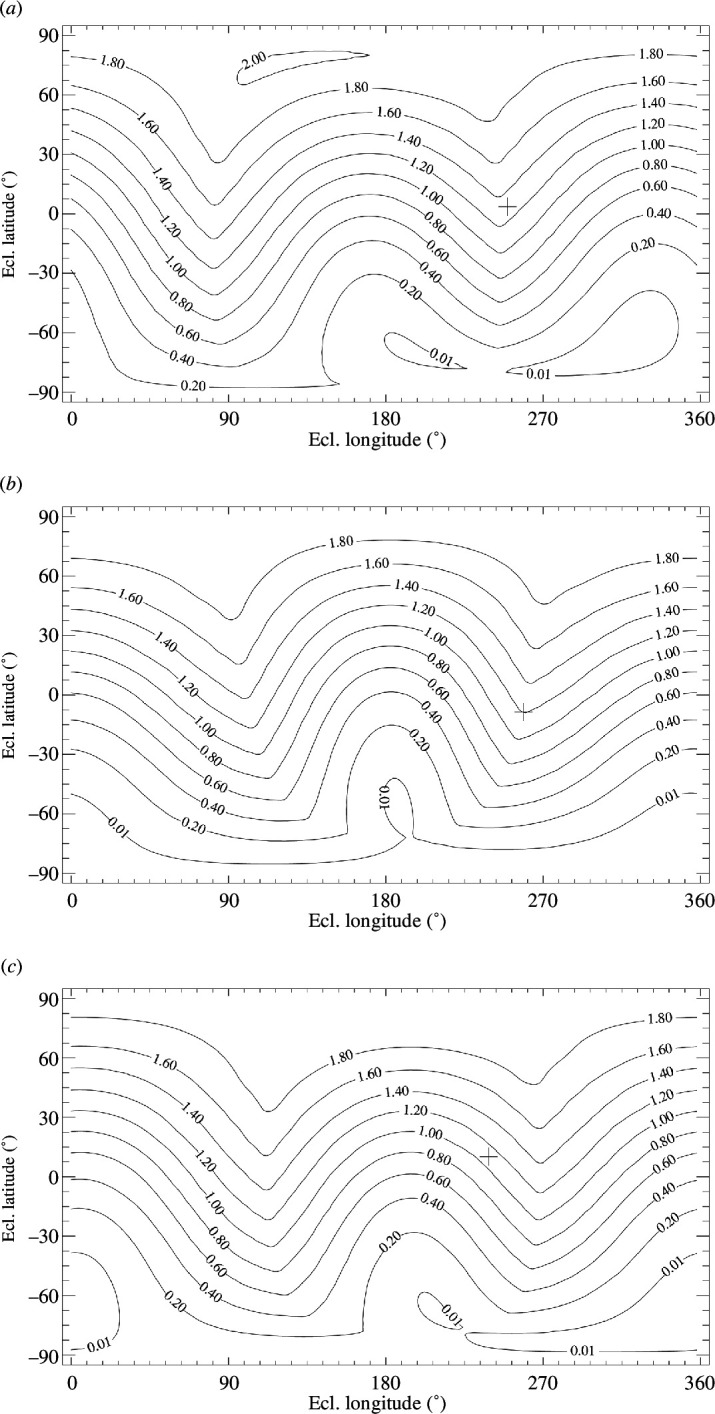
Sky maps of the Ulysses dust detector’s relative sensitivity in the solar system barycentric frame for the three dust stream detections reported in this work. We use the spacecraft position and velocity vector on the date for each stream detection for calculating the transformation between the solar system barycentric reference frame and the spacecraft frame. The ‘+’ sign denotes the direction of the stream in the solar system frame as predicted by the IMEX Streams model. *Top panel:* Stream detection on 12 March 1995, with a stream velocity of 31.8 km s^−1^ as predicted by the IMEX Streams model. *Middle panel:* Detection on 25–27 April 2001, and a stream velocity of 32.2 km s^−1^. *Bottom panel:* Detection on 16–19 May 2001, and a stream velocity of 32.1 km s^−1^.

As described above, our analysis identified three traverses in the Ulysses data by a clustering of impacts on the 3σ level, out of the 19 cometary trail traverses with predicted fluxes exceeding $10^{-3}\;m^{-2}\;day^{-1}$ . These are the trail traverses of comets 267P/LONEOS on 21 March 1995, 146P/Shoemaker-LINEAR on 29 April 2001 and 10P/Tempel 2 on 13 May 2001 (the dates refer to the predicted maximum fluxes during each trail traverse). The Ulysses measurements together with the IMEX model predictions are shown in figure 6. Skymaps for these detections are shown in figures 7 and 8 in appendix B.

The top panel in each set of diagrams in figure 6 shows the dust fluxes predicted for these three trail traverses. They are also shown in figures 2 and 4. The top panels A and C show dust fluxes predicted for two additional comets below our assigned threshold flux of $10^{-3}\;m^{-2}\;day^{-1}$ , i.e. comets 45P/Honda-Mrkos-Pajdušáková (hereafter 45P/H-M-P) and P/1999 RO28 (LONEOS). We will get back to these comets below.

The second panel in each set of diagrams shows the measured impact rates derived for particles with a restricted range in rotation angles $\text{∆}ROT_{obs}{=\pm 32}^{\circ }$ around the expected impact directions $ROT_{trail}$ of the trail particles as described in §4.1. Given that the sensitive sensor area drops to half its maximum value for impact angles $\theta =32^{\circ }$ , this is consistent with a collimated practically mono-directional stream of particles as one would expect for the cometary trails, cf. Table 2.

The third panel shows the probability that the observed particle clusterings are owing to random fluctuations, calculated from equation (4.1). Here, we considered only those particles identified in a restricted range of rotation angles $\text{∆}ROT_{obs}{=\pm 32}^{\circ }$ as in the second panel. We show the probability curves for the number of particles N , which give the highest σ . The average background dust impact rate for particles detected from these directions, X , is derived from a time interval ±15 days around the perihelion passages of Ulysses. It ranges between 0.2 and 0.3 impacts per day (table 4, column (3)).

Finally, the bottom panel in each set of diagrams displays the measured rotation angles for all particle impacts detected within the six-week intervals shown. The blue dashed box shows the range in impact time and rotation angle $\text{∆}ROT_{obs}{=\pm 32}^{\circ }$ during which trail particles were identified. These boxes contain between five and seven particles. Figure 5 shows the interplanetary trajectories and trail particle approach directions onto the spacecraft for these comets.

Figure 6 shows that for two of the three candidate trail traverses the measured times agree very well with the times predicted by the IMEX model. For the remaining third comet, 267P, the particle clustering was measured 9 days before the predicted time. This poses some doubt as to whether 267P is really the source comet of the detected particles. We therefore searched our simulation results for other potential candidate comets closer in time to the measured trail traverse but with lower fluxes. It showed that the measurement coincides very well with the trail traverse predicted for comet 45P/H-M-P. We therefore included the fluxes predicted for this comet in diagram set A of figure 6 (top panel). Similarly, in set C we included two additional comets, i.e. 45P/H-M-P and P/1999 RO28.

Again, the measured impact times agree very well with the times predicted for these trail traverses. In addition, the third panel exhibits a rather long tail in the probability lasting approximately 10 days which is much longer than the predicted crossing time for comet 10P. A check of the detection geometry showed that particles originating from these two comets were indeed detectable by the sensor. However, the predicted fluxes are one to two orders of magnitude lower than those of our preferred candidates with higher fluxes. In summary, our results indicate that we have three likely comet trail traverses identified in the Ulysses *in situ* dust data which are connected with up to five comets.

### (c) Instrument noise

So far, we have assumed that all events in the Ulysses dust dataset are owing to dust impacts and, therefore, of equal significance. Although we believe that the dataset is basically noise-free (cf. §2.2), we considered the quality classes assigned to the particles by our on-board classification scheme in order to identify potential unrecognised noise events among our trail particle candidates. It turned out that most of the big particle impacts (i.e. events in the higher amplitude ranges, AR≥2 ) were registered in the higher quality classes, i.e. they have coincident detections in two or three channels. Therefore, they are in the category of the most reliable dust impacts. The neglect of the events in the lowest quality class 0 preferentially removes the smallest particles from the dataset. For our candidate comets 45P/267 P, 146P and 10P, this leaves us with three, five and three events, respectively, which we consider very reliable dust particle impacts.

We repeated our statistical analysis with this reduced dataset. For consistency, we included only the events in the higher classes in the calculation of the background flux. With this reduced dataset, we obtained similar trail detections for comets 45P/267P and 146P as before with the full dataset.

In conclusion, the fact that the dataset reduced to the highest quality classes, i.e. to the big impact events, leads to comparable trail detections and strengthens our interpretation that Ulysses really detected true dust trail particles from these two comets. On the other hand, in the case of comet 10P, the selection of only the higher quality classes leads to the removal of four small events, and our algorithm detects no particle concentration, and thus no dust trail, anymore. Given that the lowest quality class 0 always has the highest likelihood for unrecognized noise events, it makes the detection of this third cometary trail less reliable.

We made an additional check of the potential noise contamination in the Ulysses dataset. During a few months around Ulysses’ Jupiter flyby in 1992, the dust instrument detected streams of tiny dust particles emanating from the Jupiter system [46]. The particle sizes were approximately 10 nm, and they were driven out of the Jupiter system by their strong electromagnetic interaction with the planet’s magnetosphere. The Jupiter dust streams were confirmed in 2003–2005 when Ulysses made its second flyby at Jupiter [29].

We made use of the fact that the Jupiter stream particles were detected within a range in rotation angle of approximately $180^{\circ }$ , i.e. from about half a spacecraft rotation. During the other half rotation, the sensor pointed away from the dust stream approach direction, and no stream particles were detectable (figure 2). In 2004, Ulysses detected three particularly strong dust streams on days 155, 169 and 181 (streams nos. 12–14 in table 1 of [29]), during a total time interval of 32 days. In total, 3884 dust particle impacts were detected during these three streams, and their full datasets were transmitted to Earth so that the rotation angle during the impact is known.^2^ Only one event was detected from the $180^{\circ }$ wide opposite range in rotation angle. This was an event in our second-highest quality class 2 in the lowest AR, AR = 1.

Noise events should not occur from a preferred direction, they should rather be detected over the full range of rotation angles. To this end, we assume that the one impact detected from the direction opposite to the dust streams during the 32-day interval was in reality a noise event. Furthermore, assuming that during the remaining 50% of the time when the sensor pointed in the direction of the dust streams, noise events occurred with the same rate, i.e. one event during 32 days, there may be two unrecognized noise events. Assuming that the noise environment in interplanetary space around Jupiter is not too different from the one encountered in the inner Solar System around Ulysses’ perihelion where we identified the cometary trails, this leads to a fraction of potentially unrecognized noise events in the dataset of 2/3885, i.e. less than one out of one thousand particle impacts in our dust dataset may be owing to an unrecognized noise event. In a similar analysis, we used the rotation angle of the Galileo spacecraft, which was equipped with a twin of the Ulysses dust instrument, to identify noise events in the dust dataset from the Jupiter system where the noise contamination was much stronger [33]. To conclude, we are confident that the cometary trail particle candidates we identified in our analysis are real dust impacts.

### (d) Trail particle properties

With our analysis described above we identified a total number of 19 likely trail particle impacts measured during three cometary trail traverses by the Ulysses spacecraft. We now analyse the properties of these particles—in particular impact speed and particle mass—and compare them with the predictions by the IMEX model. The results are summarized in table 4.

The analysis of the Ulysses data showed that each trail crossing took only a relatively short time period of approximately one to three days. For each traverse, we calculated mean values for the measured particle impact speeds, $\bar{v_{calib}}$ , and masses, $\bar{m_{calib}}$ , based on the instrument calibration. For a single impact, both quantities are derived from up to three measured impact charges and the corresponding rise times of the charge signals. The uncertainty of the speed calibration is typically a factor of 2 and that of the particle mass a factor of 10 [24]. Given that for some particle detections the instrument calibration did not provide speed and mass values with sufficient accuracy; however, the number of particle measurements used for this calculation was smaller than the total number of detections (cf. table 4, columns (5) and (6)). Furthermore, judging from the impact rate of particles from the interplanetary background listed in column (4), between 0 and 1 impact of a background particle that does not belong to the cometary trail has to be expected during each trail traverse. We can only roughly estimate this number from the measured background flux because we cannot distinguish whether an individual particle impact was owing to a background particle or a trail particle. Based on these considerations, we do not attempt to give uncertainties for the derived impact speeds, $\bar{v_{calib}}$ , and particle masses, $\bar{m_{calib}}$ , listed in table 4, beyond the typical uncertainties for a single measurement of a factor of 2 and 10, respectively, as given above. Keeping this in mind, the measured average impact speeds between 17 and $43\;km\;s^{-1}$ are in good agreement with the model predictions listed in table 2.

The particle masses derived from the calibrated impact speeds range from approximately $4\cdot 10^{-15}$ to $4\cdot 10^{-14}\;kg$ . For an assumed density of $1000\;kg\;m^{-3}$ , this corresponds to particle radii of approximately 1–2 µm (table 4, column (9)).

A somewhat different approach to derive the particle mass can be applied if the particle impact speed is known by other means. In this case, the calibrated impact speed derived from the rise time measurement of the charge signal is ignored and only the mass is derived from the instrument calibration for the assumed impact speed. Such a technique was successfully applied earlier to Ulysses interstellar dust measurements [27,47] and to Galileo *in situ* measurements of dust particles ejected from the Galilean moons [48,49]. The impact speed was known quite accurately from theoretical considerations in both cases. In our case of the cometary trail particles, we use the impact speed predicted by the IMEX model. Taking into account that the simulated impact speeds for each trail vary by only about 10% ( $39\;-\;45\;km\;s^{-1}$ , table 2) the derived particle masses have an uncertainty of about a factor of 3 instead of the factor of 10 uncertainty based on the calibrated speeds ( $17\;-\;43\;km\;s^{-1}$ ). This leads to particle radii which are a factor of three smaller than the radii obtained when using the calibrated speeds. On the other hand, the particles may be significantly larger if they are very porous as one has to expect for cometary particles. In any case, we have to emphasize that these values are two orders of magnitudes lower than the minimum particle sizes simulated by the IMEX model. We will get back to this issue in §5.

From the number of particle detections in each trail and the time needed by the spacecraft to traverse these trails, $t_{cross}$ , we can estimate the trail particle flux Φ :


(4.2) $$\Phi =\frac{N_{det}-N_{back}}{A\cdot t_{cross}},$$

where A is the effective sensitive area of the dust detector, and $N_{back}$ is the number of interplanetary background particles detected in the same time interval. Since Ulysses was a spinning spacecraft, we have to use the spin-averaged effective sensor area [21]. Given that the cometary streams were detectable with minimum impact angles $\theta_{min}≲20^{\circ }$ , and the uncertainties imposed by the particle identification, we use a sensitive area of $A=0.018\;m^{2}$ throughout. The resulting dust trail fluxes are given in table 4, column (13).

Finally, taking the predicted impact speed from our simulations, $v_{imp}$ , we get the dust spatial density D :


(4.3) $$D=\frac{\Phi }{v_{imp}}.$$

These values are also listed in table 4. Again, they refer to the measured particle sizes which are in the range of approximately one micrometre. These dust trail fluxes and spatial densities show a remarkable agreement within a factor of four with values we have derived from the analysis of dust trail traverses by the Helios spacecraft [16].

## 5. Discussion

Comet trails were first identified in the IRAS all-sky survey [5,50]. Subsequently, in a survey of 34 Jupiter-family comets with the Spitzer Space Telescope, at least 80% of the observed comets were associated with dust trails [6]. Comet trails were also studied with the Diffuse Infrared Background Experiment (DIRBE) on board the Cosmic Background Explorer (COBE) [51] and with ground-based observations in the visible range [2,52]. Thus, cometary dust trails are now considered a common cometary feature. Nevertheless, out of the list of five candidate comets identified in our analysis with potential *in situ* trail particle detections by Ulysses, 10P/Tempel 2 is the only one detected in these infrared surveys. It indicates that *in situ* dust measurements are more sensitive to trail detections than imaging observations.

The dust trail of 10P/Tempel 2 was extensively studied, and in the IRAS all-sky survey it was by far the most prominent such structure owing to its proximity to the Earth [50]. The derived trail particle sizes were approximately 1–6 mm, and the estimated ejection speeds from the comet nucleus were a few metres per second with ejection periods over the last few hundred years. The trail likely represents only a small fraction of the total mass that should be lost by the comet nucleus over a two-hundred-year period. Smaller particles were not detectable in the infrared observations.

Another source of information about particle sizes of cometary dust is radar observations of cometary comae. Observations with the Arecibo observatory planetary radar system showed that the coma of comet 45P/H-M-P contains particles larger than 2 cm [53], while the existence of smaller particles could not be excluded. Single dust impacts in the size range of approximately $r_{d}\approx 1-10\;\text{μ}m$ which likely originated from this comet were also identified in the dust dataset measured with the Helios spacecraft [16]. Those particle sizes are somewhat larger than the ones obtained in this work for our Ulysses detections. Interestingly, comet 45P/H-M-P, among several additional comet and asteroid candidates, may be associated with the α Capricornids meteor stream [54]. This comet was also predicted to be the source of a meteor shower in the atmosphere of Venus [55].

The IMEX model simulates relatively large particle sizes $r_{d}\geq 100\;\text{μ}m$ . This is two orders of magnitude larger than the sizes derived for our Ulysses detections ( $r_{d}\approx 1-2\;\text{μ}m$ ). However, small particles are known to exist in the ejecta cloud of a comet, following a differential power law index of approximately −4 in their size distribution [2] (and references therein), which corresponds to an index of approximately −3 for a cumulative size distribution. Their dynamical behaviour is not the same as that of particles with $r_{d}≳100\;\text{μ}m$ owing to the increasing effect of non-gravitational forces on the smaller particles. However, for sizes $10\;\text{μ}m\leq r_{d}\leq 100\;\text{μ}m$ , their orbital characteristics are sufficiently similar to those of the larger particles, but they are spatially offset owing to perturbations, as preliminary tests have demonstrated. An updated version of the IMEX Streams model for smaller particle sizes is planned, but currently not available. Nevertheless, we carried out a simple simulation of smaller particles in the range of 2−10μm that hints towards detectability in the vicinity of the trail near perihelion, even after a full revolution around the Sun.

We investigated the applicability of our simulation results to smaller particles. A detailed discussion can be found in appendix C. The radiation pressure of the Sun increases the eccentricity and semi-major axis of stream particles, but for the orbits of the four streams with likely detections, the effects remain small for particle sizes of $r_{d}≳5\;\text{μ}m$ .

Assuming a simple Parker spiral model, the solar magnetic field leads to a 22-year cycle of alternating 11-year phases of focusing and defocusing with respect to the solar equatorial plane. This is driven by the charge-to-mass ratio Q/m , which follows an inverse square dependence on the particle radius $Q/m\propto r_{d}^{-2}$ , and changes severely with particle size. We ran simple simulations assuming a Parker spiral magnetic field and injecting particles at the perihelion of the four relevant comets. We conclude that the effect of the magnetic field becomes negligible for particles $r_{d}≳10\;\text{μ}m$ , notable at $r_{d}≃5\;\text{μ}m$ and dominant at sizes $r_{d}≲3\;\text{μ}m$ on timescales of 100 years.

The Poynting–Robertson effect causes micrometre-sized particles to lose energy, circularize orbits, and eventually spiral inwards towards the Sun [56]. According to [56, eq. 7], the typical timescale for particles of 5 μm on a 2 AU circular orbit with a bulk density of $1000\;kg\;m^{-3}$ is 14 000 years and is therefore irrelevant for stream particles ejected about 150 years ago. Numerical simulations have been used to confirm that the perturbations from the Poynting–Robertson drag are small compared with the radiation pressure and electromagnetic forces on these timescales.

In order to get to a more realistic representation of the expected fluxes, we consider two cases with a lower cutoff of 5μm and 10μm , respectively. We extrapolate from the IMEX model flux of particles with sizes $r_{d}\geq 100\;\text{μ}m$ using a cumulative power-law size distribution index of −3 . This leads to an increase in total flux by a factor of 1000 in the case of a cutoff at 10μm , and a factor of 8000 for the 5μm cutoff. As seen from our simulations, in this size range a large fraction of particles remain in the stream, and we consider a lower cutoff between 5 and 10μm a realistic estimate for the expected fluxes and fluences overall. Based on the fluences listed in table 1, extrapolated fluences on the order of approximately 7–20 m^−2^ for a lower cutoff at 10μm , and 56–160 m^−2^ for a cutoff at 5 μm are predicted by the IMEX model during traverses of the trails of 267P/LONEOS, 146P/Shoemaker-LINEAR, and 10P/Tempel2. Taking into account the Ulysses detector size of $0.1\;m^{2}$ and the circular scanning pattern, this leads to estimates of 0.2–2 particle detections (10 μm cutoff), and 1.8–5 particles (5 μm cutoff). This is supported by our Ulysses measurements. In addition to this, a comparison of the IMEX model to observations of cometary meteoroid streams by [1] concluded that the model likely underestimates the true fluxes by up to an order of magnitude.

Our discussion in §4.3 showed that the neglect of small particles leads to similar detections for the trails of comets 45P/267P and 146P by our statistical algorithm as with the full dataset including the small particles but rejects the detection of comet 10P. We believe that this strengthens our conclusion that we have really detected cometary trail particles in the first two cases because the trails should preferentially contain large particles. The fraction of large particles in the trails is actually *larger* than in the entire Ulysses interplanetary dataset (here of course we ignore the small Jupiter stream particles).

Our analysis of the cometary trail particles detected by Helios indicated that the size (mass) of the impactors may be significantly underestimated, even more so if cometary particles are fluffy aggregates as implied, for example, by the results from the Rosetta mission to comet 67P/Churymov–Gerasimenko [57–59]. This is also supported by particle dynamics. Particles released at perihelion from the comets listed in table 4 have orbital eccentricities in the range e=0.54−0.65 (cf. table 5). They cannot remain in bound heliocentric orbits unless their β ratios are smaller than 0.46 and 0.18 for the lower and upper eccentricity value, respectively. This means that particles below approximately 1.5–3 µm in radius are very quickly removed from these trails and escape from the Solar System on hyperbolic trajectories. If we consider comet 45P/H-M-P with an orbital eccentricity e=0.82 , this limit increases to 5μm . For very porous particles consistent with Rosetta’s results, these boundaries are a factor of 2–3 higher.

These considerations imply that the particles belonging to the cometary trails must have been significantly bigger than the sizes derived from the instrument calibration. Thus, the chances are low that Ulysses observed rather micrometre-sized and smaller cometary trail particles, confirming earlier results from our analysis of the cometary trail particles detected by Helios [16]. This is similar to the situation when Ulysses detected Jupiter stream particles [31]. We only learned from modelling that the particles actually had nanometre sizes, i.e. in that case they were much smaller than particle sizes derived from the instrument calibration, and the *in situ* measurements performed by the Cassini Cosmic Dust Analyzer showed that the majority of the particles were NaCl condensates from Io’s volcanoes likely having a compact structure [60].

The calibration of the Ulysses dust instrument was performed with compact particles throughout [61]. An underestimation of particle masses derived from the Ulysses calibration would be consistent with recent results from dust accelerator impact experiments, indicating that porous particles generate fewer ions upon impact than their compact counterparts [62], while theoretical considerations of hypervelocity impacts of porous $SiO_{2}$ dust point in the opposite direction [63]. Nevertheless, highly porous cometary trail particles are likely not well represented by the existing instrument calibrations of impact ionization dust detectors which were performed with compact particles only. This discrepancy underlines the necessity for calibration experiments with porous projectile particles to properly calibrate future dust instruments.

From our analysis presented in §4.4, we obtained dust number densities in the meteoroid trails in the range of $2-7\;\cdot 10^{-8}\;m^{-3}$ (table 4). These values agree within a factor of four with particle densities we derived for comets 45P/H-M-P and 72P/Denning-Fujikawa in a similar analysis of the Helios dust measurements [16]. For both the Ulysses and the Helios trail traverses of these comets, the IMEX model predicts similar trail particle fluxes for 100μm and bigger particles in the range of $10^{-2}\ldots 10^{-3}\;m^{-2}\;day^{-1}$ . Even though the flux predictions by IMEX have large uncertainties, this similarity in the measured dust densities may indicate that both spacecraft sampled not too different particle size ranges during their respective trail traverses.

How do the number densities we derived for the trails compare with large-scale dust densities in the ambient zodiacal cloud? To this end, we use the new Interplanetary Meteoroid Environment Model 2 (IMEM2 [19]) for the interplanetary dust environment at Earth’s orbit. For particles larger than approximately 1μm , it gives a flux of $4\cdot 10^{-10}\;m^{-3}$ , which is up to two orders of magnitude below the dust densities we derived for the cometary trails. This value agrees within a factor of two with the fluxes predicted by the earlier model for the interplanetary dust environment developed by [64] and dust measurements performed in low-Earth orbit by the Long Duration Exposure Facility [65].

Measurements with the magnetometer instrument on board Ulysses [66] showed that the spacecraft traversed the ion tails of at least three long-period comets: C/1996 B2 (Hyakutake; 1 May 1996), C/1999 T1 (McNaught-Hartley; 19/20 October 2000) and C/2006 P1 (McNaught; January/February 2007). These tail crossings showed up by magnetic field structures in the solar wind well downstream of the respective cometary comae [20]. None of these events is in our list of potential or identified trail traverses (table 1). This does not come as a surprise given that far from the nucleus the comet’s meteoroid trail and its (ion and dust) tails are expected to fill very different, usually not overlapping, spatial volumes.

Cometary meteoroid impacts may pose significant risks for Earth-orbiting satellites and crewed spaceflight. Ref. [50] already pointed out that the impacts of such particles could potentially be a significant spacecraft hazard. In their worst-case scenario, a spacecraft with a surface area of $10\;m^{2}$ exposed to dust impacts would only have to travel 330 000 km (or less than 8 trail widths) through the trail of comet 10P/Tempel 2 before having a 1 in 3 chance of impacting a millimetre-sized particle.

For 1mm sizes, the number density of trail particles derived from the IRAS observations nearest to the comet is approximately $3\cdot 10^{-10}\;m^{-3}$ [50]. This value is three orders of magnitude lower than our value derived for approximately 1μm particles (table 4). These values are roughly consistent when taking the abundances of such differently-sized particles based on their size distribution into account, and the fact that [50] sampled preferentially the space close to the comet nucleus, while the trail traverse by Ulysses occurred far away from the nucleus.

In the past, impact detections of cometary trail meteoroids on to Earth-orbiting satellites were reported from the Highly Eccentric Orbit Satellite (HEOS) [67,68] (impacting particles could be traced back to comet Kohoutek), Explorer 46 [69] and the Long Duration Exposure Facility (LDEF [65,70]). In 1993, the Olympus-1 telecommunications satellite in geostationary orbit was thought to be hit by a Perseid meteoroid and finally lost [12]. On the Moon, impacts of meteoroid trail particles leading to increased dust densities in the circum-lunar dust environment were detected by the Lunar Atmosphere and Dust Environment Explorer (LADEE [10]), and at Mercury, the Messenger spacecraft observed sharp rises in the plasma density in the planet’s exosphere which were likely related to impacts of approximately 10 centimetre-sized meteoroids on to the planet’s surface [71,72].

For future crewed missions to other planets, meteoroid impacts will likely become an increasingly severe issue because those spacecraft will become increasingly larger, and long flight times will be required which will further increase the probability for hazardous meteoroid hits. In the future, the impact hazard should already be considered during the analysis and design phase of interplanetary missions in order to optimize their trajectories and reduce hazardous cometary trail traverses as much as possible [73].

We applied the IMEX model to predict cometary stream traverses also by other missions, including BepiColombo [41], Parker Solar Probe [74] and MMX [42]. The statistical analysis method successfully applied to the identification of trail particles in the Ulysses data in this work can be applied to identify cometary trail particle impacts in the dust data of these and other missions equipped with *in situ* dust instruments in the future.

Last but not least, we also performed IMEX simulations for the DESTINY^+^ (Demonstration and Experiment of Space Technology for INterplanetary voYage with Phaethon fLyby and dUst Science) mission to the asteroid (3200) Phaethon [75,76] scheduled for launch in 2025 [77].

## 6. Summary and conclusions

We re-analysed the full dataset of measurements obtained with the *in situ* dust detector on board the Ulysses spacecraft between 1990 and 2007, searching for impacts by cometary meteoroid trail particles. This analysis was motivated by the positive detection of single trail particle candidates in the *in situ* dust dataset measured in the 1970s with the Helios spacecraft in the inner Solar System [16].

The identification of dust trail particle impacts in these two datasets only became possible with the advent of modern fast computers which allow the application of sophisticated and comprehensive modelling techniques. To this end, we applied the IMEX Dust Streams in Space model developed by [1,18] which simulates recently created cometary meteoroid streams in the inner Solar System, to make predictions for cometary trail traverses by the Ulysses spacecraft. Without this powerful tool, the identification of cometary dust trail particle impacts in the data would not have been possible, given the low fluxes of dust particles in the trails. In fact, the measurement of cometary trail traverses was one of the major scientific goals for the Ulysses dust investigations from the beginning, but they became reality only 30 years after the launch of the spacecraft.

We identified three likely cometary trail traverses of the Ulysses spacecraft in the *in situ* dust impact data on 12 March 1995, 25–27 April 2001 and 16–19 May 2001. The source comets are 10P/Tempel 2, 146P/Shoemaker-LINEAR and 267P/LONEOS, and possibly 45 P/H-M-P and P/1999 RO28 (LONEOS). Using the Ulysses measurements in combination with the IMEX simulations, we found spatial densities of dust particles with approximately micrometre radii in these cometary trails to be approximately $2-7\cdot 10^{-8}\;m^{-3}$ . Our analysis confirms earlier results that trail particles are likely detectable with an *in situ* dust impact detector when the spacecraft traverses such a dense cometary dust trail. This emphasizes the possibility to analyse celestial bodies remotely, without the necessity to fly a spacecraft close to or even land on the source objects.

## Figures and Tables

**Figure 9.**
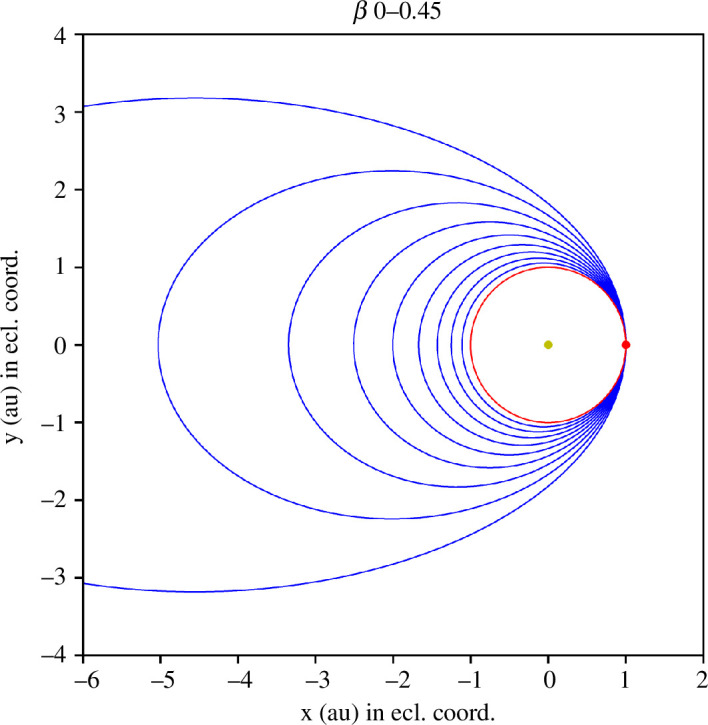
Comparison of Keplerian orbits with different values for β , starting with the same initial position and velocity. In order to illustrate the effects of β , only solar gravity and radiation pressure are taken into account for this representation, and the particles remain on closed orbits. The red dot marks the initial position for all orbits. It is located at a distance of 1 AU from the Sun (yellow dot) at the circular velocity of 29.8 km s^−1^ pointing upward. The red orbit shows a circular orbit with β=0 , and the blue orbits of increasing sizes correspond to increasing values for β in steps of 0.05 for β = 0.05, 0.10, 0.15, 0.20, 0.25, 0.30, 0.35, 0.40 and 0.45.

**Figure 10.**
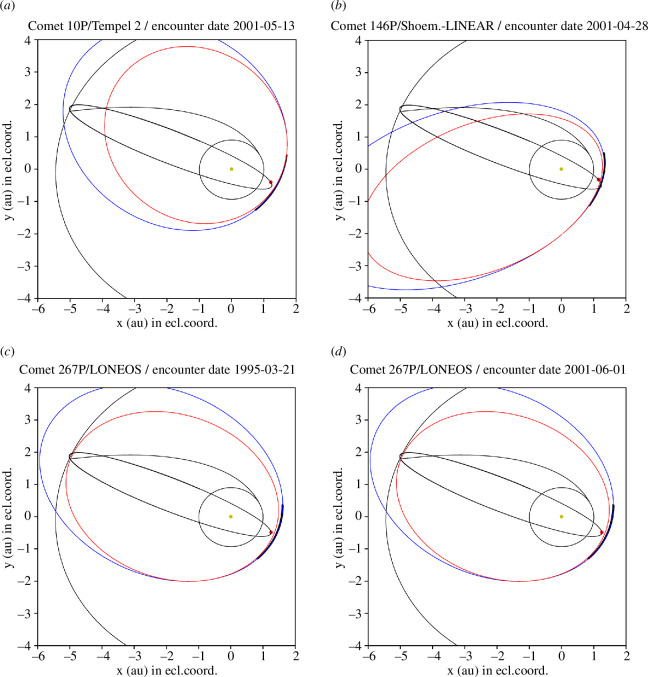
View of the encounters of Ulysses with simulated comet streams. The blue orbit shows the orbit of particles leaving the comet at perihelion with β = 0.07 for particles on a Keplerian orbit with a size of 5 μm , the red orbit corresponds to a similar population of particles but with β = 0 (i.e. following the comet’s orbit), and the simulated particle positions within 1 AU of Ulysses are overlaid in black on the blue orbit. Furthermore, we show the Sun in yellow, and the orbits of Ulysses, Earth and Jupiter in black.

**Figure 11.**
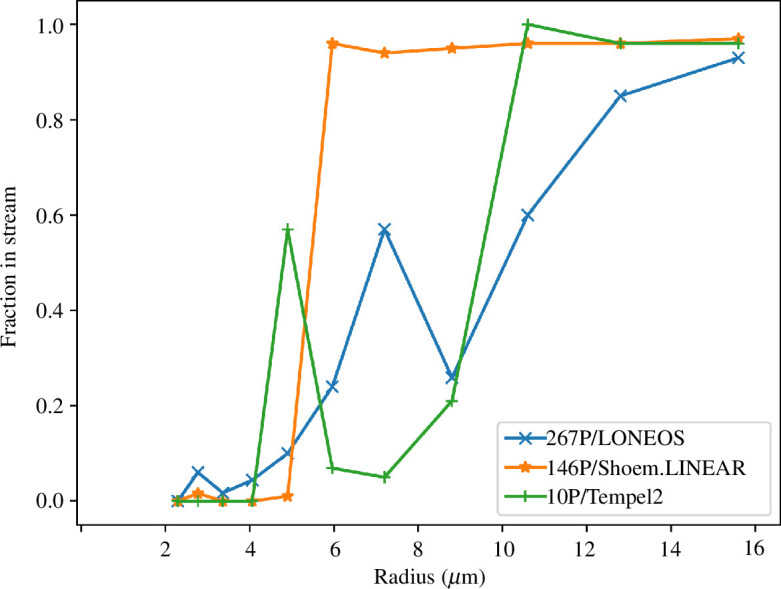
Fraction of simulated particles remaining within 1.5 ⋅10^6^ km of the initial orbit after an integration time of 150 years under the forces of solar gravity, radiation pressure, and Parker solar magnetic field. The two encounters with comet 267P/LONEOS differ by less than 0.01 and are therefore not distinguished in the plot. The plot clearly shows that particles <5 μm largely escape the stream on these timescales, for particles of 5 μm < r_d_ < 10 μm a significant fraction remains inside the stream, and for sizes > 10 μm the large majority stays close to the initial orbit. This supports the possibility of observing cometary stream particles > 5 μm with *in situ* instruments.

## Data Availability

The Ulysses dust data we use have been analysed under different objectives before, and the results have been published in various scientific papers. The original data are available via the Planetary Data System.

## Footnotes

1 Note that this title will soon be taken over by the Student Dust Counter on board the New Horizons spacecraft.

2 Approximately one thousand additional particles were detected, but their datasets could not be transmitted to Earth because of the limited data transmission capabilities of Ulysses. These particles were only counted by the dust instrument and their impact direction is not known.

## Appendix A. Orbital elements of candidate comets

Table 5 lists the orbital elements of the comets considered in our analysis.

**Table 5.** Orbital elements of comets discussed in this paper from the JPL small body database (ssd.jpl.nasa.gov) (rounded to two digits).

comet (1)	spice ID (2)	epoch (yyyy-mm-dd) (3)	period (yr) (4)	*q* (AU) 5)	*e* (6)	*i* ${(}^{\circ })$ (7)	ω ${(}^{\circ })$ (8)	Ω ${(}^{\circ })$ (9)	$t_{Perihelion}$ (yyyy-mm-dd) (10)
10P/Tempel 2	10 00 094	2012-03-20.0	5.37	1.42	0.54	12.03	195.59	117.80	2010-07-04.70
21P/Giacobini-Zinner	10 00 032	2017-11-06.0	6.55	1.01	0.71	32.00	172.81	195.41	2018-09-10.27
25D/Neujmin 2	10 00 064	1927-03-21.0	5.43	1.34	0.57	10.64	193.70	328.72	1927-01-16.22
47P/Ashbrook-Jackson	10 00 003	2011-03-03.0	8.34	2.80	0.32	13.05	357.83	356.98	2009-02-01.39
51P/Harrington	10 00 038	2015-10-05.0	7.16	1.70	0.54	5.42	269.28	83.70	2015-08-12.69
51P/Harrington-A	10 00 363	2000-05-13.0	6.78	1.57	0.56	8.66	233.59	119.19	2001-06-05.69
69P/Taylor	10 00 092	2016-04-17.0	7.67	2.28	0.41	22.04	343.67	104.85	2019-03-18.42
70P/Kojima	10 00 053	2011-01-23.0	7.05	2.01	0.45	6.60	2.02	119.26	2007-10-05.11
146P/Shoemaker-LINEAR	10 00 311	2013-06-29.0	8.11	1.43	0.65	23.09	317.07	53.45	2016-06-29.57
162P/Siding Spring	10 00 525	2010-03-23.0	5.33	1.23	0.60	27.82	356.31	31.24	2010-03-08.42
163P/NEAT	10 00 521	2006-11-20.0	7.06	1.93	0.48	12.50	348.05	103.35	2005-02-01.35
189P/NEAT	10 00 400	2008-10-14.0	4.98	1.17	0.60	20.40	15.30	282.20	2007-07-25.94
238P/Read	10 01 676	2008-05-30.0	5.62	2.36	0.25	1.27	325.62	51.63	2011-03-12.35
267P/LONEOS	10 02 412	2007-09-25.0	5.97	1.34	0.60	5.37	96.86	245.25	2006-09-02.93
304P/Ory^b^	10 02 989	2001-07-13.0	5.84	1.38	0.57	2.76	329.66	60.68	2008-10-19.69
383P/Christensen^a^	10 02 415	2007-11-10.0	6.54	1.36	0.61	11.87	128.06	213.64	2006-08-30.23
P/2008 O3 (Boattini)	10 02 511	2008-08-28.0	23.42	2.50	0.70	32.27	340.98	47.57	2008-06-03.08
45P/Honda-Mrkos-Pajdušáková	10 00 045	2017-02-14.0	5.26	0.53	0.82	4.25	326.28	88.98	2016-12-31.27
P/1999 RO28 (LONEOS)	10 00 271	1999-09-20.0	6.63	1.23	0.65	8.19	219.86	148.45	1999-10-02.34

^a^ Other designation: P 2006 R1 Siding Spring.

^b^ For the Spice ID 1002989 the JPL Horizons database gives the name 304P/Ory while the IMEXStreams model gives P/2008 O3 (Boattini).

## Appendix B. Skymaps

Here, we provide sky maps of the Ulysses dust detector’s sensitivity for the detection geometry of the three streams reported in this work. The directions shown in the maps refer to the apex direction of a hypothetical dust flow, they are *antiparallel* to the dust’s velocity vector.

First, we show the sensitive area of the detector projected onto the sky, measured in the spacecraft frame of reference, taking into account the rotation of the Ulysses spacecraft ([Fig AF7] figure 7). The path the detector axis takes in the sky reaches the full sensitive area of 1000 cm^2^, other areas further away from the sensor axis have lower dust sensitivities, according to the detector’s sensitivity profile. The observation geometry is taken into account for the three events of high significance as discussed previously.

Second, we show the relative sensitivity of the detector for dust sources of constant velocity but the direction varying over the celestial sphere, with respect to the frame of the solar system barycentre (figure 8). The maps are shown in ecliptic longitude and latitude for the apex of a dust flow of constant velocity, but directions vary all over the sky. The sensitivity is given relative to the detector’s sensitive area, so if the spacecraft were stationary with respect to the solar system, the maximum relative sensitivity would be 1 for a dust flow along the instrument axis. However, in the direction of the spacecraft’s motion, the apparent dust flow can be enhanced by almost a factor of 2 and reduced to approximately 0 in the opposite direction. This means that the coordinates correspond to the direction where a hypothetical detector that is at rest with respect to the solar system needs to point in order to measure dust from that direction.

The barycentric reference frame is independent of the spacecraft’s motion relative to the dust. All the effects related to the spacecraft and its detector are in the sensitivity contours only.

The contours mapped in figure 8 correspond to the maximum sensitive area Ulysses reaches for a given direction owing to its scanning pattern on the sky, enhanced by a factor determined by the relative velocity of Ulysses with respect to the dust flow. When the spacecraft moves into the dust flow, the effective sensitive area increases. When it moves away from the flow, the flux decreases. Owing to this effect, the effective sensitive area in these maps can be larger than the geometric area of the sensor.

## Appendix C. Dynamics of small particles

### C.1. Overview

The IMEX Streams model was conceived as an engineering model to assess the hazard to spacecraft travelling in interplanetary space. For this purpose, the inclusion of particle sizes <100μm was not required, and at the time the model was created, their contribution to the risk was deemed negligible. However, for the purpose of interpreting *in situ* dust observations in the Solar System, where typical particle sizes are <5μm , this is a major shortcoming that would best be addressed by extending the model towards lower sizes down to the 1−10μm range. This is foreseen for a future upgrade, but currently, simpler ways must be found to understand the applicability of the simulation results to smaller particles.

To this end, we used the orbital data of the three most promising candidate comets from this work and integrated the orbits of test particles ejected at perihelion. This leads to a general understanding of the relative effects of non-gravitational forces acting on these particles and their deviation from unperturbed orbits, as well as the dependence on particle size. The typical size distribution of cometary grains reaches down to micrometre-sized particles, owing to a typical differential power-law $\propto r_{d}^{-4}$ , with an index of −4 for the differential size distribution, and an index of −3 for the cumulative distribution.

We quantify the results by determining two parameters: the fraction of particles remaining inside a tube of $1.5\cdot 10^{6}$ km around a reference orbit, using either the comet’s orbit or the orbit of a particle with a given radiation pressure β for reference. We also calculate the effect of β in terms of the overall distance between the disturbed orbit and the original comet’s orbit near the point of detection by Ulysses.

The orbits used in the detailed investigation correspond to four encounters with three comet trails, namely, 267P/LONEOS (2 flybys of Ulysses, on 21.03.1995 and 01.06.2001), 146P/Shoemaker-LINEAR (flyby on 29.04.2001), and 10P/Tempel 2 (13.05.2001). For the conversion between mass and radius, we used a bulk density of $1000\;kg\;m^{-3}$ throughout.

### C.2. Forces

#### C.2.1. Solar gravity and radiation pressure

The Sun’s force of gravity and radiation pressure follow the same $r^{-2}$ dependence, thus their relative effect in interplanetary space can be quantified by $\beta =F_{rad}/F_{grav}$ , leading to an effective combined force of $F_{eff}=(1-\beta )F_{grav}$ . The radiation pressure essentially reduces the attractive potential of the Sun, and ‍ β>1 leads to a repulsive potential. Particles with 0<β<1 therefore move on Keplerian orbits but in a gravitational potential reduced by a factor of (1−β) .

When a dust particle with β>0 is ejected from a comet at a negligible relative velocity, the particle starts with the same position and velocity as the comet. The comet does not experience radiation pressure (i.e. β=0 ), and therefore the particle, owing to its β , moves on a larger orbit with an increased semi-major axis and eccentricity, and the ejection point being a tangent point to the comet’s orbit (figure 9). If β is small enough, the dust particle remains on a bound orbit. This is typically the case for cometary particles with $r_{d}≳5\;\text{μ}m$ at β≳0.06 , but particles with sizes of 1 μm and smaller can reach larger values of β and get ejected from the Solar System on hyperbolic orbits.

As most stream particles are ejected from the comet near its perihelion, the resulting dust streams are somewhat enlarged over the parent comet’s orbit but remain close to the comet’s orbit in the inner Solar System (figure 10). In order to quantify this deviation, we calculated the distance between the β=0 case and the β≠0 case for the four possible Ulysses stream encounters near the detection point. For the reported Ulysses stream candidates, the shift owing to β≠0 is $3\cdot 10^{6}$ km for 10P/Tempel 2, $5\cdot 10^{6}$ km for 146P/Shoemaker-LINEAR and $11\cdot 10^{6}$ km for 267P/LONEOS.

#### C.2.2. Lorentz force

In order to quantify the effects of the solar magnetic field, we simulated particles using a Parker magnetic field, as described in Ref. [78]. Particles were injected at the comets’ perihelion at the position and velocity of the comet, with an additional velocity component of $100\;m\;s^{-1}$ with a random direction. After an integration time of 150 years, more than 90% of the particle trajectories stay within a tube of $1.5\cdot 10^{6}$ km around the original comet orbit for particles with $r_{d}\geq 10\;\text{μ}m$ , and 48–85% of the particle trajectories for particles of $r_{d}=7.2\;\text{μ}m$ , and 1–60% for a particle size of 5μm , when observed near the detection point of the Ulysses spacecraft. The ranges given for the percentages refer to the variation among the three different comet trails. Particles of $r_{d}≲3\;\text{μ}m$ do not stay within the given tube.

We used tubes for β=0 with β taken from a β -curve derived from geometric considerations, which is a good approximation for larger particles. Owing to the cross-section increasing with $A_{cross}=\pi r_{d}^{2}$ , and the mass with $m_{d}=(4\pi /3)\rho r_{d}^{3}$ , the effect of the radiation pressure decreases as $\beta \propto A_{cross}/m_{d}\propto r_{d}^{-1}$ .

#### C.2.3. Poynting–Robertson effect

In the context of long-term dynamical evolution of dust particles in the Solar System, the Poynting–Robertson (PR) effect plays an important role [56]. It acts as a drag force slowly dissipating a particle’s orbital energy owing to a relativistic interaction with the solar radiation, leading to a circularisation of elliptical orbits, and then to an inward spiral of the orbit. However, the typical timescales for particles of $1\;\text{μ}m<r_{d}<100\;\text{μ}m$ on circular orbits at 2 AU for the PR drag to become relevant are on the order of $3\cdot 10^{3}\ldots 3\cdot 10^{5}$ years [56, eq. 7], and thus outside of the scope of this discussion. We also verified using numerical integration that perturbations owing to the PR drag for particles on orbits inside the three comet trails remain small ( $\ll 1.5\cdot 10^{6}\;$ km, our criterion for particles remaining inside the trail) for particle sizes >5μm on timescales of 150 years.

### C.3. Discussion

Among the forces presented above, only the combined solar gravity/radiation pressure and the Lorentz force play a significant role in the context of micrometre-sized young stream particles. Under our simple assumptions, we find that the Lorentz force leads to a disruption of streams for particle sizes ≲3μm , but for $r_{d}≳5-7\;\text{μ}m$ , the majority of stream particles remain close to the orbit predicted by the effects of β and gravity alone within $1.5\cdot 10^{6}$ km (figure 11).

These particles stay on orbits that are almost closed on timescales of 100 years and return to their point of ejection. Near the perihelion, the particle densities therefore remain high within a tube around the comet’s orbit. Far away from the perihelion though, the effect of β≥0 leads to a marked increase in aphelion distance, and the stream becomes more diffuse for a range of β , as illustrated in figure 9.

For the reported Ulysses stream candidates, the shift owing to β≠0 remains at $3\cdot 10^{6}$ km for 10P/Tempel 2, $5\cdot 10^{6}$ km for 146P/Shoemaker-LINEAR, and $11\cdot 10^{6}$ km for 267P/LONEOS. The first two values are well within the limits of the typical stream distances obtained from the IMEX Streams model and do not lead to a significant change in the dynamics of small particles compared with the 100 μm streams. The larger distance for 267P/LONEOS corresponds to a Ulysses flight time of 3 days. Depending on the flyby geometry and the width of the trail, this can lead to a significant change in the detectability of small particles for Ulysses.

Note that this is not a complete simulation of the stream candidates identified in the Ulysses data. However, we argue that the dynamical behaviour of smaller particles down to about 5 μm on timescales of 100 years is still similar enough to the dynamics of the larger particles to allow for useful estimates using the IMEX Streams model.
